# aPKC regulates apical localization of Lgl to restrict elongation of microridges in developing zebrafish epidermis

**DOI:** 10.1038/ncomms11643

**Published:** 2016-06-01

**Authors:** Renuka Raman, Indraneel Damle, Rahul Rote, Shamik Banerjee, Chaitanya Dingare, Mahendra Sonawane

**Affiliations:** 1 Department of Biological Sciences, Tata Institute of Fundamental Research, Colaba, Mumbai 400005, India; 2 SASTRA University, Tirumalaisamudram, Thanjavur 613402, India

## Abstract

Epithelial cells exhibit apical membrane protrusions, which confer specific functions to epithelial tissues. Microridges are short actin protrusions that are laterally long and form a maze-like pattern in the apical domain. They are widely found on vertebrate squamous epithelia including epidermis and have functions in mucous retention, membrane storage and abrasion resistance. It is largely unknown how the formation of these laterally long actin projections is regulated. Here, we show that antagonistic interactions between aPKC and Lgl–regulators of apical and basolateral domain identity, respectively,–control the length of microridges in the zebrafish periderm, the outermost layer of the epidermis. aPKC regulates the levels of Lgl and the active form of non-muscle myosinII at the apical cortex to prevent actin polymerization-dependent precocious fusion and elongation of microridges. Our data unravels the functional significance of exclusion of Lgl from the apical domain in epithelial cells.

## Abstract (web summary)

Squamous epithelia present actin-rich microridges on the apical surface, but the mechanism of their formation is not known. Here the authors show that, in zebrafish epidermis, the exclusion of the basolateral regulator Lgl from the apical domain by atypical protein kinase C prevents precocious elongation and fusion of microridges.

## Graphical abstract

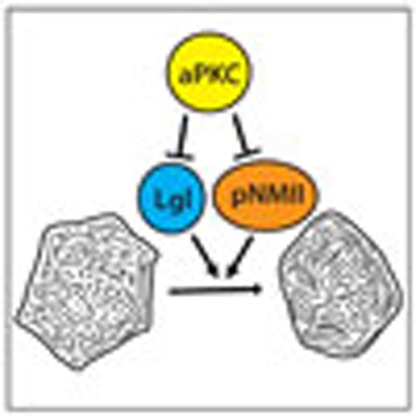

In amniotes, several kinds of epithelial cells exhibit apical membrane protrusions supported by the actin cytoskeleton. One of the classic examples of apical projections is microvilli in the intestine, kidney and the brain ventricles having secretory and absorptive functions. Microridges–another kind of apical actin protrusions–are widely found on vertebrate squamous epithelia such as epidermis, cornea, oral mucosa, vaginal epithelium and urinary bladder^1^,^2^,^3^. Unlike microvilli, which are tall-cylindrical projections, microridges are short but laterally long structures, forming a labyrinth in the apical domain. The proposed function of microridges include mucous retention, membrane storage and abrasion resistance^4^,^5^. It is largely unknown how microridges are formed and whether their formation is under the control of mechanisms that regulate cell polarity in the epithelial cells.

Cell polarity along the apical-basal axis is regulated by four major complexes, Par3-Par6-aPKC, Crumbs-Stardust-PatJ, Lgl-Dlg-Scrib and Yurt-Coracle-NrxIV, which are highly conserved across phyla^6^,^7^. In epithelial cells and in neuroblasts, Par3-Par6-aPKC complex is localized to the apical domain, whereas Lgl-Dlg-Scrib complex is localized to the basolateral domain^6^,^8^,^9^,^10^. In Drosophila epithelia, loss-of-function analyses of the Par3-Par6-aPKC complex results in a loss of apical membrane identity, mislocalization of basolateral membrane markers, failure to assemble adherens and septate junctions and loss of epithelial organization^11^,^12^,^13^,^14^,^15^,^16^. Atypical protein kinase C (aPKC), a serine/threonine kinase localized at the apical domain, contributes towards the maintenance of apical identity by phosphorylation and removal of Lgl from the apical domain^16^. The loss of aPKC function results in loss of apical membrane identity as basolateral components mislocalize to the apical domain^9^,^15^,^17^. The apical aPKC and basolateral Lgl thus act antagonistically to regulate many aspects of epithelial cell polarity and asymmetric division of neuroblasts^9^,^15^,^16^,^18^,^19^.

Vertebrates have two paralogues of *lgl*, namely *lgl1* and *lgl2*. In mice, *lgl1* function is essential for the maintenance of cell polarity in the neuroepithelial cells^20^, whereas *lgl2* functions in precise branching morphogenesis during placental development^21^. In zebrafish, *penner/lgl2* function is necessary for the formation of hemidesmosomes and to prevent hyperplasia of the basal epidermis^22^,^23^,^24^. Lgl has been proposed to execute its cellular functions by regulating polarized exocytosis or by actomyosin-based mechanism^25^,^26^,^27^. Drosophila Lgl as well as vertebrate Lgl1 physically interact with non-muscle myosinII (NMII)^8^,^28^,^29^,^30^, which is an actin-based motor protein. The formation of a complex between NMII and Lgl depends on Lgl's phosphorylation status, which is regulated by aPKC^31^,^32^,^33^. In epithelial cells, Lgl regulated NMII activity controls actomyosin contractility during early stages of cell–cell contact formation and planar polarization^34^,^35^. Furthermore, in migrating cells, Lgl is known to regulate the localization of NMII and focal adhesion morphology to optimize cell migration and promote protrusion formation^30^,^32^.

So far, it has remained unclear whether the elimination of Lgl from the apical domain by aPKC has any functional consequence in vertebrate epithelia. We show that the aPKCλ function is critical in restricting the length of microridges, whereas Lgl promotes their elongation. Our study points to a novel and unknown functional antagonism between Lgl and aPKCλ in regulation of apical F-actin protrusions in the vertebrate epidermis. Mechanistically, aPKC regulates levels of Lgl and active NMII at the apical cortex to inhibit actin polymerization-dependent precocious fusion and elongation of microridges. Our analyses unravel the functional significance of removal of Lgl by aPKC at the apical domain in vertebrate epithelial cells. We propose that the formation of apical projections is under regulation of the mechanisms that control cell polarization.

## Results

### aPKC prevents precocious elongation of apical microridges

**Figure 1:**
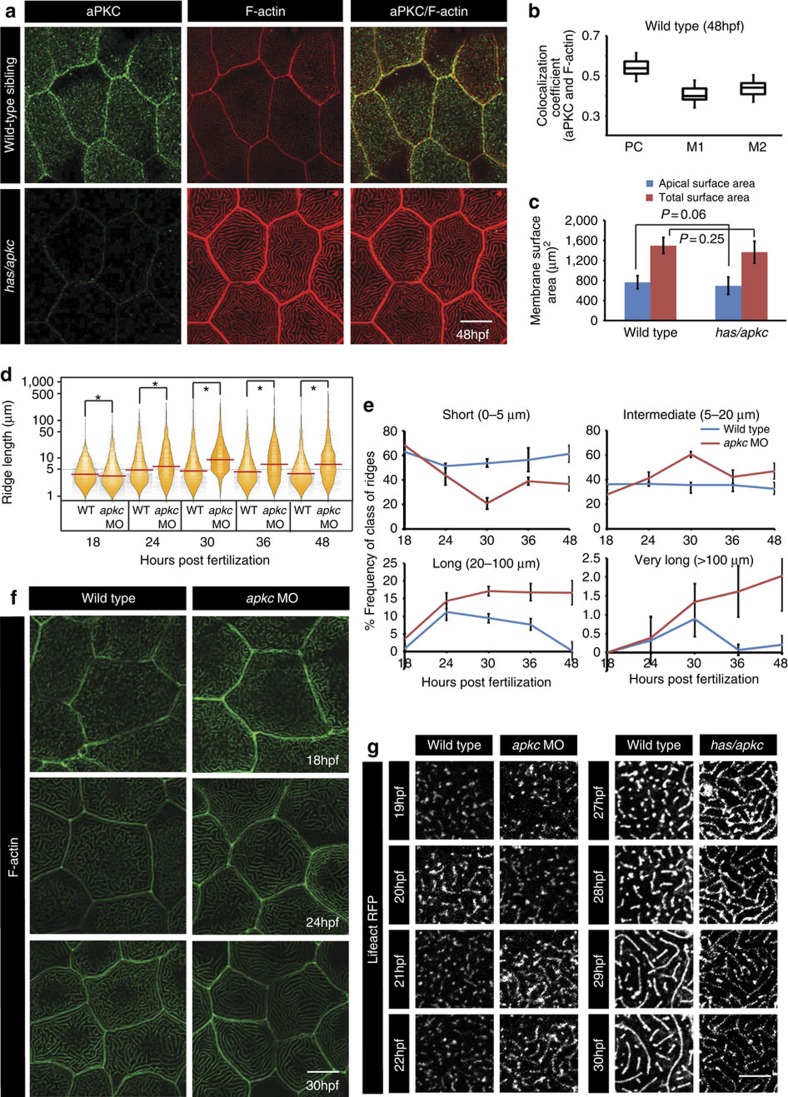
*has/apkc* function is important for restricting the length of microridges by preventing their precocious elongation. Immuno-colocalization using anti-aPKC antibody and phalloidin, which labels F-actin, in wild type and *has/apkc* mutant at 48 hpf (**a**) followed by colocalization coefficient analysis by Pearson's (PC) method and Manders' overlap for aPKC with F-actin (M1) and F-actin with aPKC (M2) at 48 hpf in wild-type embryos (**b**). Graphical representation (**c**) of apical surface area and total cell surface area in wild type and *has/apkc* mutant at 48 hpf. Visualization of the distribution of ridge lengths and medians in wild-type and aPKC-deficient embryos at various developmental time points using bean plots (**d**). The frequency distribution of ridges (**e**) in short (0–5 μm), intermediate (5–20 μm), long (20–100 μm) and very long (>100 μm) categories during development. Quantification in **d** and **e** are based on phalloidin stainings performed in wild-type and *apkc* morphants (**f**). Time-lapse imaging of microridges in clones expressing lifeActRFP under CMV promoter in wild-type and *apkc* morphants during 19–22 hpf and in *has/apkc* siblings (wild type) and mutants during 27–30 hpf (**g**). Error bars in **c** and **e** indicate s.d., whereas square brackets represent comparison. Probability values ‘*P*' are for the Student's *t*-test. Asterisks in the bean plot (**d**) represent significant difference in median values at *P*<0.05 as calculated by pairwise multiple comparison procedures using Dunn's Method. Scale bars in **a**,**f** and **g** correspond to 10 μm.

In vertebrates, there are two aPKC isoforms namely aPKCλ/ι and aPKCζ. The *apkcλ/ι* mutant in zebrafish, *heart and soul (has)*, shows defects in the morphogenesis of retina, neural tube and intestine due to improper formation and maintenance of adherens junctions^36^. We found that aPKC localizes to the apical domain of peridermal cells at 48 h post fertilization (hpf) (Fig. 1a; Supplementary Fig. 1a). Pearson's coefficient as well as Manders' overlap coefficient revealed substantial colocalization between aPKC and F-actin at the apical microridges. For this analysis, we used E-cadherin localization with apical F-actin as a negative control and Ezrin localization with F-actin as a positive control (Fig. 1b; Supplementary Fig. 1b,d). This apical localization of aPKC is diminished in *has/apkcλ/ι* mutants as well as in *apkcλ/ι* morphants (*apkc* MO)^36^ and resulted in longer and fewer microridges (Fig. 1a; Supplementary Fig. 1c,e). Such long microridges appear in the wild-type peridermal cells only after 72 hpf suggesting their precocious elongation in aPKCλ/ι deficient embryos (henceforth called just *apkc* or aPKC). E-cadherin and ZO1 staining revealed that there was no effect on formation or localization of adherens and tight junctions in the periderm of *has/apkc* mutants (Supplementary Fig. 1f). To test whether microridges in the *has/apkc* mutants become long to accommodate excess apical membrane, the *has/apkc* mutant line was crossed to the Tg(cldnB:lynEGFP) line^37^ to visualize the cell membrane of peridermal cells. Quantification of the apical as well as total surface area of peridermal cells did not reveal significant difference between the *has/apkc* mutant and wild-type siblings (Fig. 1c). Thus, increased microridge length in *has/apkc* mutants is not a consequence of increased apical surface area or impaired cell junction formation.

To gain insights into how microridges elongate over time, they were imaged in developing wild-type and aPKC-deficient embryos (Fig. 1f). Since knockdown of *apkc* recapitulates the microridge phenotype (Supplementary Fig. 1c), an antisense morpholino was used for this analysis^36^. From embryos stained for F-actin, the microridge number and microridge perimeter was quantified. The perimeter is governed by the length in the absence of appreciable changes in the microridge width. For the sake of simplicity, perimeter is referred to as ridge length in the text and all the figures. The visualization of data by bean plot and the analysis of medians revealed that though the ridges tend to be slightly shorter at 18 hpf, there is significant shift in the length distribution towards longer ridges from 24 hpf onwards in aPKC-deficient embryos as compared with wild-type embryos (Fig. 1d). Consistently, the frequency distribution analysis revealed that while the number of short ridges decreases, the number of ridges having perimeter length above 5 μm increases in *apkc* morphants during development (Fig. 1e). As compared with control, the total number of ridges is significantly lower in aPKC-deficient embryos (Supplementary Fig. 1e). This decrease in total number of ridges in the *apkc* morphant indicated that the shorter ridges fuse to form long ridges. To test this, we performed time-lapse imaging of peridermal cells expressing LifeActRFP^38^. Under given live imaging conditions, we did not observe appreciable increase in microridge length till 27 hpf in the wild-type embryo (Fig. 1g, Supplementary Videos 1 and 2). The length of microridges starts to increase effectively from 27 hpf onwards due to increase in effective fusion events (Fig. 1g; Supplementary Videos 3 and 4). Intriguingly, microridge length starts to increase precociously in *apkc* morphants from 20 to 22 hpf onwards by fusion of smaller microridges. These microridges continue to stay long but are dynamic at later time points (Fig. 1g; Supplementary Videos 5, 6, 7, 8). Movies with higher temporal resolution at 21 hpf indicate that microridges appear as punctae in wild-type epidermal cells which do not undergo elongation, whereas microridges fuse to form longer ridges in the *apkc* morphant (Supplementary Videos 9, 10, 11, 12).

To conclude, aPKC restricts the length of apical microridges in the peridermal cells of the zebrafish epidermis during development.

### aPKC controls apical localization of Lgl in the periderm

**Figure 2:**
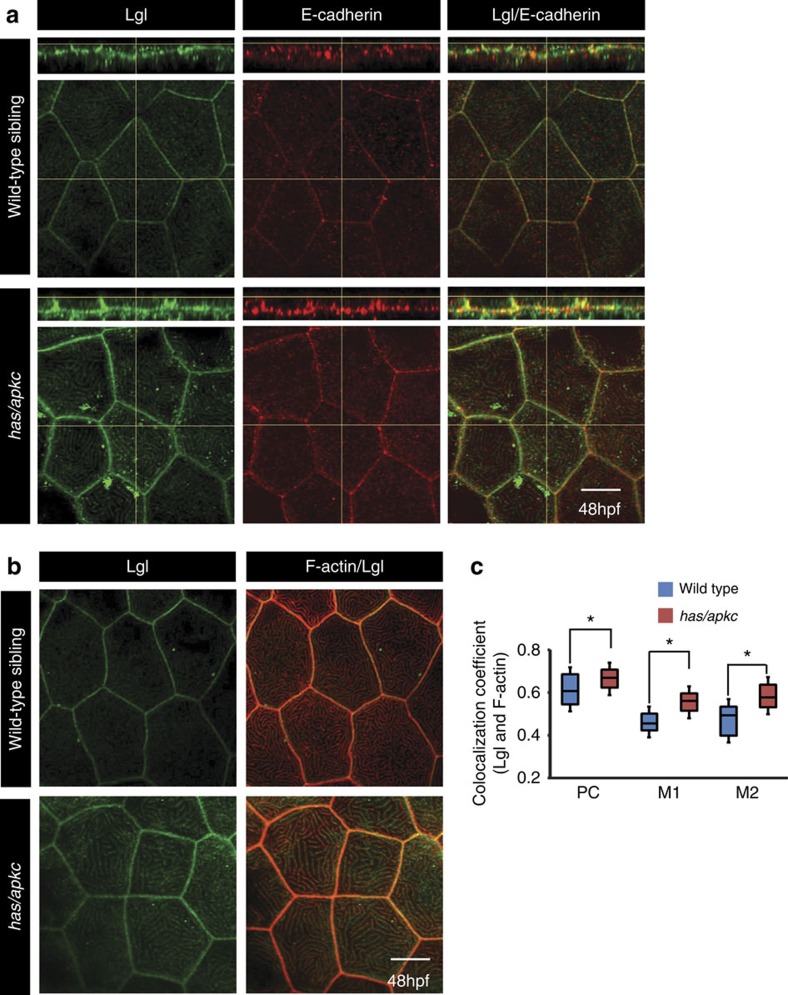
The levels of apical Lgl increase in the apical domain of the head peridermal cells in *has/apkc* mutants. Immunostaining using anti-Lgl2 and E-cadherin antibodies (**a**) in wild-type and *has/apkc* mutant embryos. Immuno-colocalization of Lgl and F-actin in the apical domain of wild-type and *has/apkc* mutant embryos at 48 hpf (**b**). Colocalization coefficient analysis between Lgl and F-actin by Pearson's (PC) method and Manders' overlap for Lgl with F-actin (M1) and F-actin with Lgl (M2) at 48 hpf in wild-type and *has/apkc* mutant embryos (**c**). Asterisks in **c** indicate significant difference at *P*<0.001 by Student's *t*-test. Scale bar in **a** and **b** is equivalent to 10 μm.

In polarized cells, Lgl is localized to the basolateral domain where it is required for establishing domain identity^8^,^26^,^39^. This exclusive basolateral localization of Lgl is achieved by selective removal of Lgl from the apical domain by its phosphorylation by aPKC^8^,^40^,^41^. Therefore, we asked whether Lgl localization is altered in the peridermal cells of the *has/apkc* mutant embryos. Immuno-localization studies using anti-Lgl2 antibody revealed that at 48 hpf a minor fraction of Lgl localizes to the apical domain in wild-type, while *has/apkc* mutants exhibit slightly higher apical localization (Fig. 2a). At the apical domain, Lgl co-localizes with F-actin at the microridges in wild-type embryos (Fig. 2b). Pearson and Manders' coefficient analysis confirmed that this colocalization is significantly higher in *has/apkc* mutants as compared with wild-type embryos (Fig. 2b,c). Temporal analysis indicated that Lgl localizes to the microridges at 24 hpf in both wild-type and aPKC-deficient embryos (Supplementary Fig. 2a). As development progresses, a minor fraction of Lgl is retained at the microridges in wild-type embryos, whereas Lgl levels increase in *has/apkc* mutants, suggesting that aPKC function is essential for controlling the levels of apical Lgl in the peridermal cells (Supplementary Fig. 2b).

### Lgl promotes elongation of microridges in the apical domain

**Figure 3:**
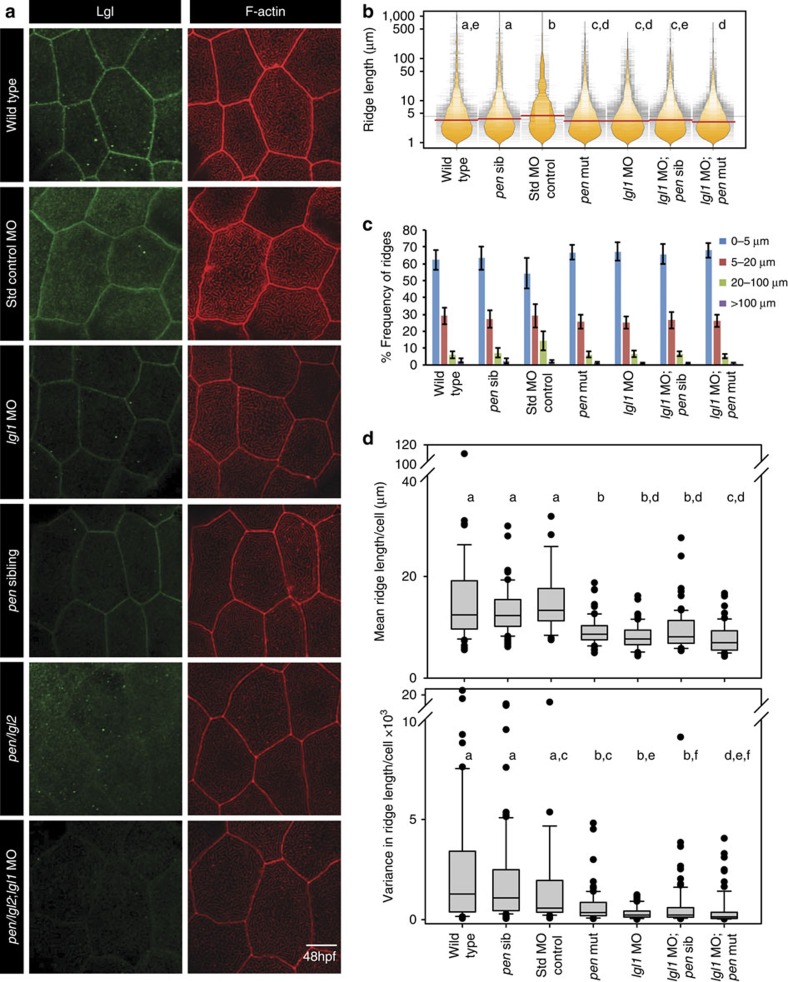
Lgl functions in the elongation of microridges in the apical domain. Immuno-localization of Lgl and F-actin in the apical domain of peridermal cells in given genetic conditions or morpholino injections at 48 hpf (**a**). Visualization of the distribution of ridge lengths and medians at 48 hpf, in various genetic conditions mentioned, using bean plots and horizontal lines, respectively (**b**) followed by estimation of percentage frequency distribution of ridges in short (0–5 μm), intermediate (5–20 μm), long (20–100 μm) and very long (>100 μm) categories (**c**). Estimation of mean and variance in ridge length for individual cells and comparison of the distributions of the means and variances across various genotypes using box-whisker plots (**d**). In **b** and **d** the distributions denoted by same alphabet or distributions that share an alphabet in common (when denoted by multiple alphabets) do not differ significantly (Dunn's multiple comparisons test, *P*-value<0.05; see Methods section for details). In **c** s.d. is shown by error bars. Scale bar in **a** is equivalent to 10 μm. MO, morphant/morpholino; Std, standard.

We asked whether *lgl1* or *lgl2* function is essential for the elongation of microridges in the periderm of wild type or *has/apkc* mutant embryos. To perform the loss-of-function analysis, we used previously published *lgl1* morpholinos^42^,^43^ or *penner (pen)/lgl2* mutant^22^. Rescue experiments revealed that the morpholino directed against the translational start site–but not UTR–gives specific phenotype in the epidermis (see Supplementary Note and Supplementary Fig. 9 for details). The *lgl1* morphant displayed reduction in Lgl localization when anti-Lgl2 antibody was used suggesting that the antibody cross-reacts to Lgl1 in zebrafish. As published before, the *pen/lgl2* mutants do not exhibit appreciable staining for Lgl2 at 5 days post fertilization when anti-Lgl2 antibody was used^24^. In absence of any detectable phenotype, we used this antibody to screen for *lgl2* mutants at 48 hpf. Despite the large variations in microridge lengths, the decrease in Lgl1 levels or absence of Lgl2 resulted in subtle but significant shift towards increase in shorter ridges as revealed by the bean plot and the decrease in medians (Fig. 3a,b). Consistently, the frequency distribution analysis revealed an increase in short (0–5 μ) ridges and reduction in the ridges that are longer than 100 μ (Fig. 3c). In addition, estimation of mean and variance for individual cells, followed by analysis of their distribution was done. We reasoned that the mean ridge length for a cell would decrease if there is a decrease in the number of longer ridges and increase in shorter ones. On the contrary, the mean ridge length for a cell would increase with increase in the number of longer ridges and decrease in shorter ridges. Similarly, the variance for each cell would either decrease or increase if the range of ridge lengths–from short to long ridges–gets smaller or larger, respectively. To maintain the robustness in the ridge length analysis in all subsequent experiments involving various genetic conditions, we followed these multiple ways of data analysis to assess the effects on ridges. The means and variances show significant decrease in Lgl1 and Lgl2 deficient cells (Fig. 3d). This analysis further suggested a decrease in the ridge lengths in absence of Lgl1/Lgl2 function. Interestingly, combined loss of both *lgl1 and pen/lgl2* function did not result in further decrease in the length of microridges in most of the embryos (Fig. 3a–d).

**Figure 4:**
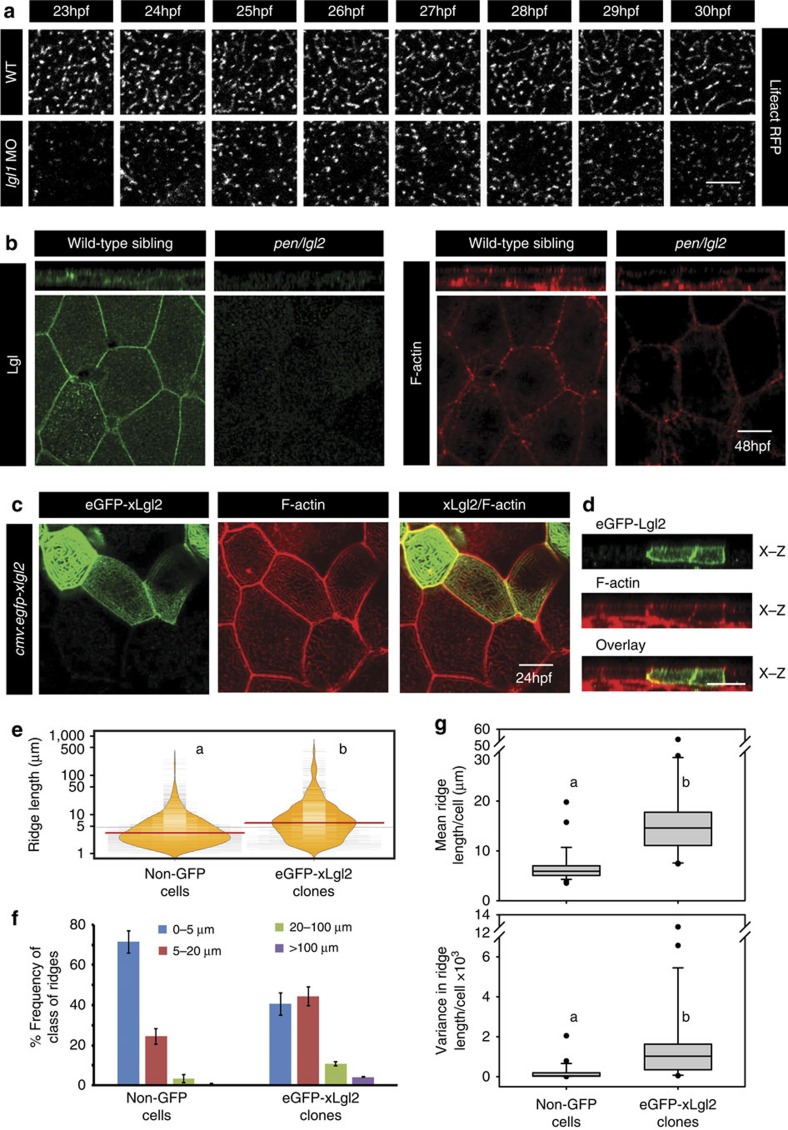
Lgl regulates the length of microridges by promoting their fusion. Live time-lapse imaging of microridge elongation (**a**) in clones expressing lifeActRFP under CMV promoter in wild-type and *lgl1* morphant embryos during 23–30 hpf. Immunolocalization of Lgl and F-actin at the basolateral domain in wild-type and *pen/lgl2* mutant at 48 hpf (**b**). Confocal images of immunostaining using anti-GFP antibody and phalloidin in wild-type embryos-injected with eGFP-xLgl2 construct under CMV promoter at 24 hpf (**c**) and their orthogonal sections (**d**). Visualization of the distribution of the ridge lengths and medians in eGFP-xLgl2 expressing clones and surrounding non-GFP cells using bean plots (**e**). The frequency distribution of ridges in short (0–5 μm), intermediate (5–20 μm), long (20–100 μm) and very long (>100 μm) categories (**f**). The box-whisker plots (**g**) represent the distributions of means and variances per cell in eGFP-xLgl2 expressing clones and surrounding non-GFP cells. Data presented in **e**–**g** is based on ridge-length measurements done on phalloidin stainings performed in the eGFP-xLgl2 injected embryos at 24 hpf. In bean plots (**e**) and box-whisker plots (**g**) the alphabets ‘a' and ‘b' represent significant difference in median values at *P*<0.05 (pairwise multiple comparison using Dunn's Method). Error bars in **f** represent the s.d. Scale bars in **a**–**d** correspond to 10 μm.

Consistent with the decrease in ridge length in Lgl deficient embryos, live time-lapse imaging of microridges revealed that in contrast to wild-type, ridges fail to fuse and continue to remain shorter in the *lgl1* morphant peridermal cells during 23–30 hpf (Fig. 4a; Supplementary Videos 13, 14, 15, 16). Reduction in Lgl2 levels also resulted in decreased F-actin levels at the basolateral domain (Fig. 4b) indicating Lgl's role in mediating F-actin assembly at the basolateral domain. There was no effect on formation or localization of tight junctions and adherens junctions in epidermal cells, which had reduced Lgl1 levels (Supplementary Fig. 2c). To test whether Lgl function is sufficient to increase microridge length in wild-type embryos, we increased Lgl levels by overexpression of Xenopus *lgl2* (Fig. 4c). Clonal analysis followed by quantification and analyses of ridge length distribution revealed that as compared with the surrounding peridermal cells, Lgl2 overexpression results in significant shift in length distribution towards longer, contiguous microridges similar to the *has/apkc* mutant (Fig. 4c,e–g). In addition, increase in Lgl leads to increased F-actin at the basolateral domain (Fig. 4d). Lgl2 overexpression did not have any overt cell adhesion or polarity phenotype as assessed by aPKC and E-cadherin staining (Supplementary Fig. 2d).

### Microridge elongation in *has/apkc* depends on Lgl levels

**Figure 5:**
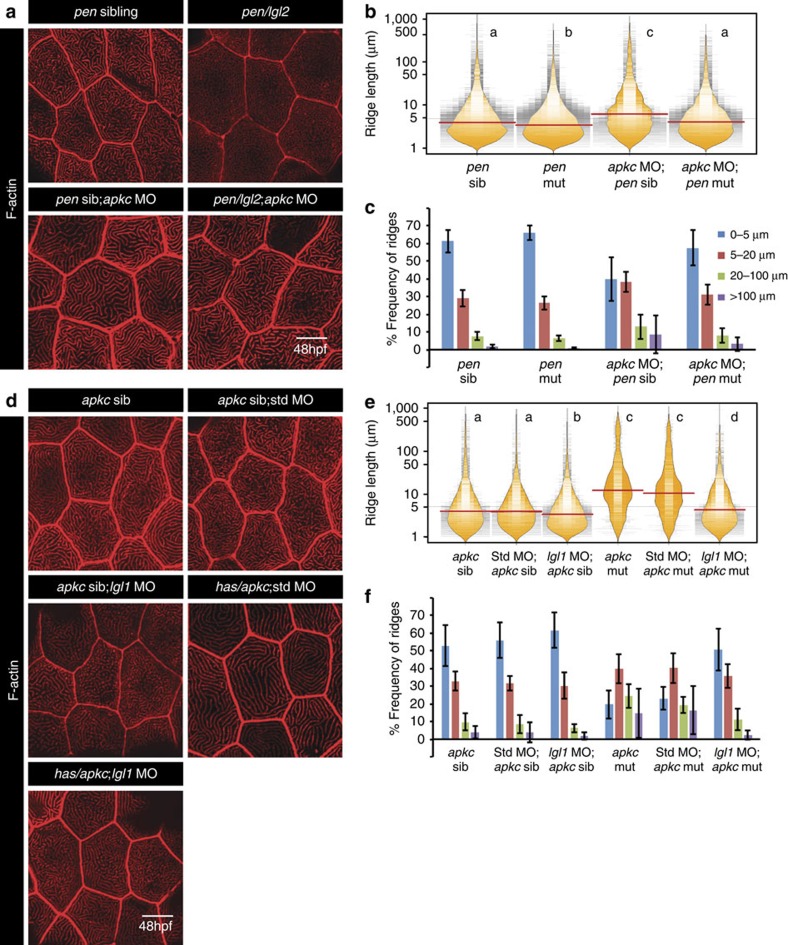
Microridge elongation in aPKC-deficient embryos occurs in an Lgl-dependent manner. Phalloidin stainings (**a**,**d**) at 48 hpf in genotypes mentioned. Ridge length estimation followed by visualization of the spread and frequency of their lengths and medians using bean plots (**b**,**e**). The frequency distribution of ridges (**c**,**f**) in short (0–5 μm), intermediate (5–20 μm), long (20–100 μm) and very long (>100 μm) categories across the above mentioned genotypes. In **b** and **e** the distributions sharing the same alphabet do not differ significantly (Dunn's multiple comparisons test, *P*-value<0.05). Error bars in **c** and **f** represent the s.d. Scale bars in **a** and **d** corresponds to 10 μm.

**Figure 6:**
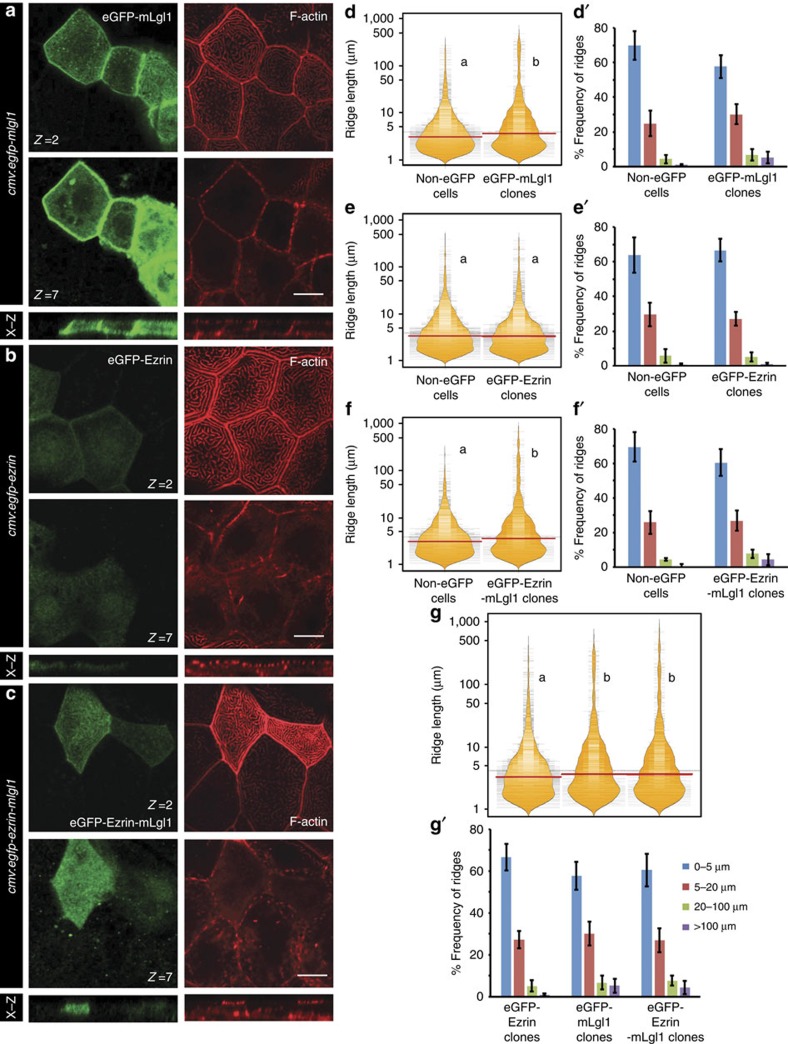
Apically localized Lgl promotes an increase in the microridge length. Confocal sections at the apical (*z*=2) and basolateral level (*z*=7) of the peridermal cells stained (**a**–**c**) for GFP and F-actin at 30 hpf in wild-type embryos injected with eGFP-mLgl1 (**a**) eGFP-Ezrin (**b**) and eGFP-Ezrin-mLgl1 (**c**) under CMV promoter along with their corresponding orthogonal sections. Visualization of the distribution of ridge lengths and medians–estimated from clones expressing eGFP-mLgl1 (**d**) eGFP-Ezrin (**e**) and eGFP-Ezrin-mLgl1 (**f**) and their corresponding non-GFP controls using bean plots. Comparison between the ridge lengths exhibited by clones expressing eGFP-Ezrin, eGFP-mLgl1 and eGFP-Ezrin-mLgl1 (**g**). The frequency distribution of ridges in short (0–5 μm), intermediate (5–20 μm), long (20–100 μm) and very long (>100 μm) categories for clones expressing eGFP-mLgl1 (**d′**), eGFP-Ezrin (**e′**) and eGFP-Ezrin-mLgl1 (**f′**) along with their corresponding non-GFP controls. The comparison between frequency distributions observed in clones expressing eGFP-Ezrin, eGFP-mLgl1 and eGFP-Ezrin-mLgl1 (**g′**). Quantifications in (**d**–**g**) and (**d′**–**g′**) are based on phalloidin stainings performed at 30 hpf in the embryos injected with the above mentioned eGFP constructs. Note the minimal localization of eGFP-Ezrin-mLgl1 and eGFP Ezrin to the basolateral cortex as compared with eGFP-Lgl1. The distributions represented by two different alphabets in **d**–**g** show significant difference at *P*<0.05 (Dunn's multiple comparisons test). Error bars in (**d′**–**g′**) represent the s.d. Scale bars in **a**–**c** correspond to 10 μm.

We hypothesized that in the absence of aPKC function, increased Lgl levels at the apical domain cause microridges to become longer. To test the hypothesis, we reduced Lgl levels–using *penner/lgl2* mutant or knockdown of *lgl1*–in aPKC-deficient embryos. The bean plots, frequency distributions and distribution of means as well as variances clearly revealed a shift in the length distribution towards shorter ridges, suggesting rescue of the aPKC phenotype (Fig. 5a–f; Supplementary Fig. 3a,b). These data suggest that in *has/apkc* mutants, microridge elongation occurs in an Lgl-dependent manner. To further confirm that the apically localized Lgl promotes an increase in the microridge length, we clonally expressed enhanced green fluorescent protein (eGFP)-Ezrin tagged mouse Lgl1 (mLgl1) in the peridermal cells. Ezrin is an essential component of the apical actin-based projections^44^ and localizes to the microridges (Supplementary Fig. 1d). The eGFP-Ezrin-mLgl1 gets targeted to the apical domain and results in an increase in the ridge length as compared with the surrounding cells that do not express this fusion construct. The overexpression of eGFP-Ezrin does not lead to increase in microridge length. Similar to eGFP-Ezrin-Lgl1, overexpression of eGFP-mLgl1 also results in longer ridges in comparison to the neighbouring cells (Fig. 6; Supplementary Fig. 3c–f).

These lines of evidence indicate that Lgl1/2 promotes formation of long ridges presumably by increasing the fusion of shorter ridges. Furthermore, Lgl has the ability to regulate F-actin formation throughout the cortex but aPKC controls Lgl levels at the apical domain to restrict microridge length.

### NMII activity is required for microridge elongation

**Figure 7:**
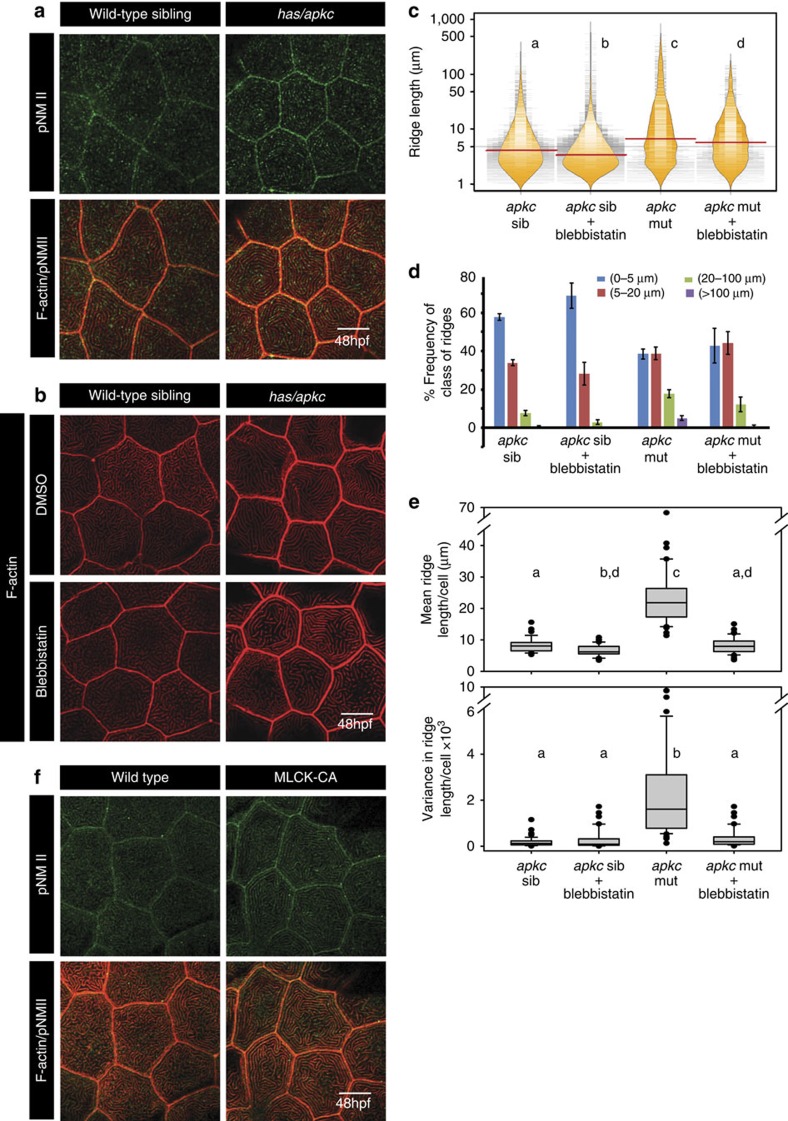
Activity of non-muscle myosinII (NMII) is essential and sufficient for elongation of microridges. Immuno-colocalization using anti pNMII antibody and phalloidin in wild-type and *has/apkc* mutant at 48 hpf (**a**). Wild-type and *has/apkc* mutant treated with 10 μM Blebbistatin at 48 hpf and stained using phalloidin to visualize F-actin (**b**). Graphical representation of the distribution of ridge lengths and medians of Blebbistatin treated and control embryos at 48 hpf using bean plots (**c**). The same data is presented as percentage frequency distribution of ridges in short (0–5 μm), intermediate (5–20 μm), long (20–100 μm) and very long (>100 μm) categories (**d**). Distribution of means and variances of ridge lengths (**e**) for individual peridermal cells- in wild-type sibling and *has/apkc* mutant treated with DMSO and Blebbistatin at 48 hpf–presented in box-whisker plots. Immuno-colocalization using anti pNMII antibody and phalloidin staining in wild-type embryos and embryos over-expressing constitutively active MLCK at 48 hpf (**f**). In **c** and **e** the distributions sharing the same alphabet do not differ significantly (Dunn's multiple comparisons test, *P*-value<0.05). In ((**e**) top) the distributions for *apkc* sib and *apkc* sib+Blebbistatin are considered statistically significantly different at *P*=0.058. Error bars in **d** are for the s.d. Scale bars in **a**,**b** and **f** are equivalent to 10 μm.

Lgl was originally identified as a cytoskeletal protein, which forms a complex with NMII and gets released from the complex on activation by aPKC kinase activity^28^,^29^,^31^. NMII is a motor protein having actin cross-linking and contractile properties^45^. It is a major component of stabilized actin structures like stress fibres^45^,^46^. The clonal expression of NMII heavy chain or light chain in peridermal cells revealed that both the chains localize to the microridges (Supplementary Fig. 4a). Despite the fact that both the chains would be overexpressed under CMV promoter, the clones did not exhibit any noticeable increase in the microridge length (Supplementary Fig. 4a). The quantification followed by distribution analysis revealed that while medians exhibit slight but significant shift on the higher side, neither frequency distribution nor analysis of means and variances revealed any significant increase in the microridge length suggesting negligible effect of the overexpression (Supplementary Fig. 4b–d). Immuno-localization using phospho-myosinII (pNMII) antibody–which detects phosphorylation of the regulatory light chain of NMII at the serine 19 residue–revealed that pNMII co-localizes with F-actin at the microridges in both wild-type and *has/apkc* mutants at 48 hpf (Fig. 7a). Temporal analysis indicated that pNMII localizes to the microridges as early as 20–24 hpf in both wild-type and aPKC-deficient embryos (Supplementary Fig. 5a). There was a quantifiable increase in localization of pNMII to the apical domain in *has/apkc* mutants at 36 hpf (Supplementary Fig. 5a,b).

To determine whether NMII activity is required for formation and elongation of microridges, NMII function was inhibited using chemical inhibitor Blebbistatin, which suppresses MyosinII ATPase activity and actin–myosin cross-linking^47^. Treatment of embryos with Blebbistatin for 1.5 h at a concentration of 10 μM–low enough to keep the cortical actin and cellular architecture intact–resulted in increase in shorter ridges in wild-type embryos at 48 hpf. In *has/apkc* mutant, the treatment resulted in a shift in the length distribution towards shorter ridges as compared with the untreated mutants (Fig. 7b–e). Blebbistatin also inhibited elongation of microridges when the treatment was done at early stages while the microridges are growing by fusion–during 20–22 hpf in aPKC-deficient and between 27 and 30 hpf in wild-type embryos (Supplementary Fig. 5c,d). This analysis suggests that NMII function is required for elongation of ridges and maintenance of their length in wild-type as well as in aPKC-deficient embryos.

To check whether function of active NMII is sufficient to increase microridge length, we overexpressed the constitutively active form of myosin light chain kinase (MLCK-CA), which is a specific activator of NMII function. MLCK-CA messenger RNA (mRNA) injected embryos showed obvious increase in pNMII staining and phenocopied the *has/apkc* mutant phenotype at 48 hpf (Fig. 7f). We conclude that the phosphorylated or active form of NMII is necessary and sufficient to increase and maintain the microridge length.

### Lgl and active NMII cooperate to regulate microridge length

**Figure 8:**
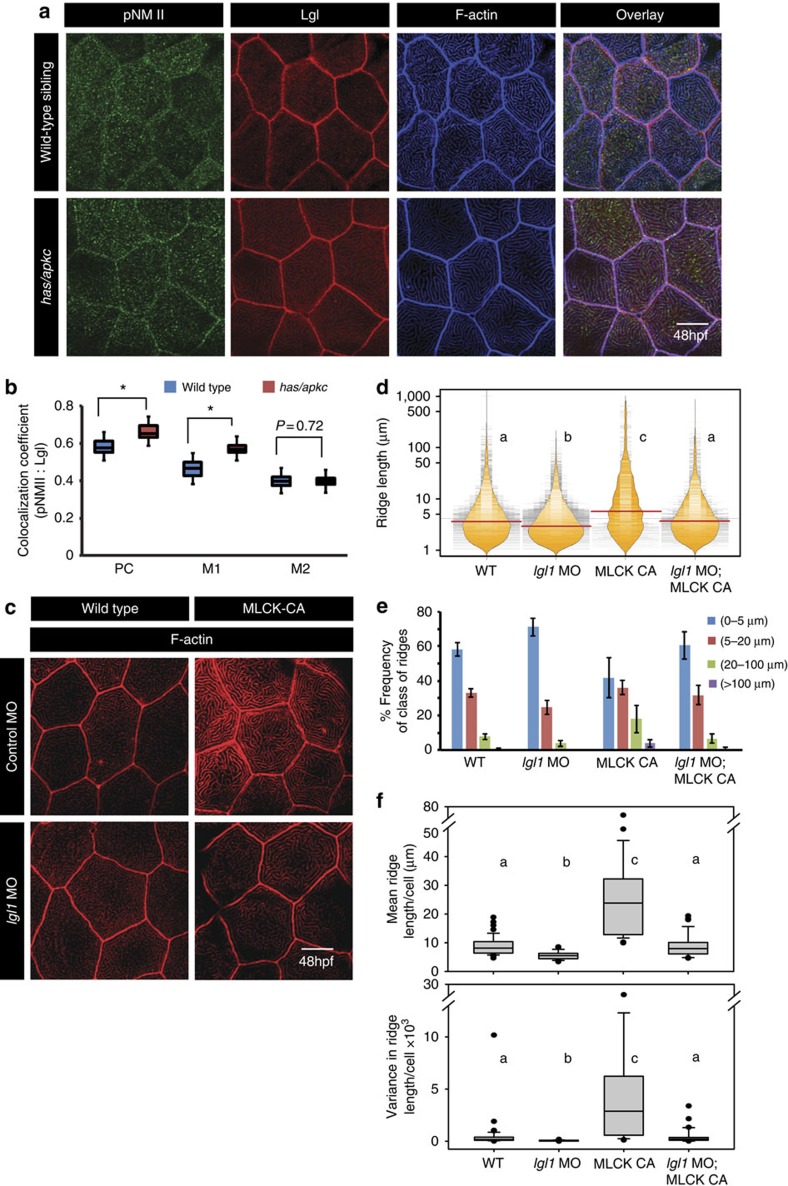
Both Lgl and active MyosinII are required to build long ridges. Immuno-colocalization using anti pNMII, anti-Lgl2 antibodies and phalloidin (**a**) in wild- type and *has/apkc* mutant at 48 hpf. Graphical representation (**b**) of colocalization coefficients for pNMII and Lgl by Pearson's method and Manders' overlap of pNMII with Lgl (M1) and Lgl with pNMII (M2) in wild-type and *has/apkc* mutant embryos at 48 hpf. Phalloidin stainings (**c**) in embryos injected with control morpholino, MLCK-CA+control morpholino, *lgl1* morpholino and MLCK-CA+*lgl1* morpholino at 48 hpf, followed by ridge length measurements and visualization of distribution of ridge lengths and medians using bean plots (**d**). The same data is presented as percentage frequency distribution of ridges in short (0–5 μm), intermediate (5–20 μm), long (20–100 μm) and very long (>100 μm) categories (**e**). The box-whisker plots (**f**) represent distributions of means and variances of ridge lengths for individual cells from various genetic conditions. In **d** and **f**, the distributions sharing the same alphabet do not differ significantly (Dunn's multiple comparisons test, *P*-value<0.05). Error bars in **e** are for the s.d. Asterisks in **b** indicate significant difference at *P*<0.001 by Student's *t*-test. Scale bars in **a** and **c** is equivalent to 10 μm.

Lgl genetically and physically interacts with NMII and regulates its activity and distribution by binding to the coiled-coil rod domain of the heavy chain of NMII (refs 25, 26, 28, 29, 30, 34, 35). Immunostainings followed by analysis of colocalization coefficients showed that Lgl and pNMII show increased colocalization at the microridges in *has/apkc* mutants as compared with wild type siblings at 48 hpf (Fig. 8a,b). To check whether both Lgl and active NMII are essential to regulate microridge length, *lgl1* knockdown was achieved in embryos over-expressing MLCK-CA. Down regulation of *lgl1* in MLCK-CA overexpression background rescued the long microridge phenotype caused due to increased NMII activity (Fig. 8c–f). Similarly, overexpression of MLCK-CA did not yield longer microridge phenotype in *pen/lgl2* mutant (Supplementary Fig. 6a–d). Furthermore, Lgl2 overexpression resulted in mild increase in pNMII levels (Supplementary Fig. 7a). Consistently, inhibition of NMII activity by Blebbistatin in Lgl2 over-expressing peridermal cells resulted in smaller microridges at 24 and 30 hpf (Supplementary Fig. 7b,c). We conclude that both Lgl and active NMII cooperate in zebrafish peridermal cells to regulate microridge length.

### F-actin polymerization in microridge elongation and maintenance

**Figure 9:**
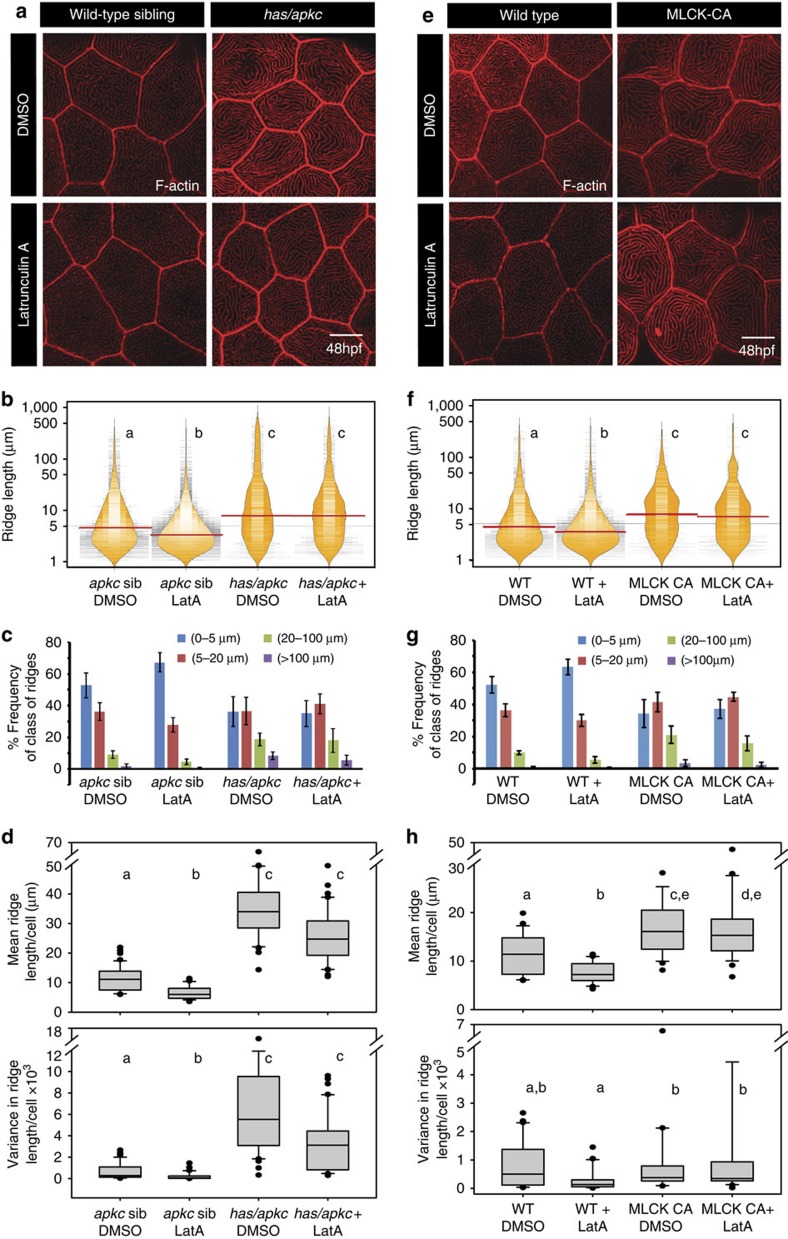
The loss of aPKC function and overexpression of MLCK-CA results in formation of Latrunculin resistant stable microridges. Phalloidin stainings of *has/apkc* sibling *and* mutant embryos (**a**) and wild type and MLCK-CA overexpression embryos (**e**) treated with Latrunculin A. Visualization of the spread and frequency of ridge lengths and medians from the given genotypes and treatments using bean plots (**b**,**f**). Estimation of percentage frequency distribution of ridges in short (0–5 μm), intermediate (5–20 μm), long (20–100 μm) and very long (>100 μm) categories (**c**,**g**). Estimation of cell wise means and variances of ridge lengths based on phalloidin stainings (**d**,**h**). In **b**,**d**,**f** and **h**, the distributions sharing the same alphabet do not differ significantly (Dunn's multiple comparisons test, *P*-value<0.05). The two distributions in ((**h**) top), WT DMSO and MLCK-CA+LatA, are different at *P*=0.061. Error bars in **c** and **g** are for the s.d. Scale bars in **a** and **e** are equivalent to 10 μm.

We further investigated the contribution of F-actin polymerization in the elongation and maintenance of ridges under various genetic conditions. Microridges are formed of F-actin filaments organized perpendicular to the plasma membrane^1^, similar to other actin-based projections^48^. We treated *has/apkc* mutants, MLCK-CA embryos and wild-type embryos at 48 hpf with Latrunculin A. In addition, we also treated 5-day-old larvae with Latrunculin because the microridges are long and convoluted in 5-day-old larvae, similar to 2-day-old *has/apkc* mutants. Latrunculin associates with actin monomers and prevents them from getting incorporated into filaments^49^. Thus, Latrunculin treatment would lead to net increase in actin depolymerization. Treatment of 48 hpf wild-type and *has/apkc* sibling embryos with a brief pulse of Latrunculin resulted in shortening of microridges. After treatment, the occurrence of short ridges increases significantly in wild-type and *has/apkc* sibling at 48 hpf, as revealed by the bean plot, frequency distribution and analysis of means and variances. These data suggest that actin polymerization is required to maintain the length of these structures. Surprisingly, microridges in *has/apkc* mutants and MLCK-CA over-expressing peridermal cells remained relatively unaffected by Latrunculin treatment at 48 hpf (Fig. 9a–h). However, both *apkc* morphants and MLCK-CA over-expressing embryos were susceptible to Latrunculin treatment when the ridges are actively growing in these two conditions, leading to shorter ridges (Supplementary Fig. 8a). Similarly, in 5-day-old wild-type larvae and in Lgl2 overexpression condition, Latrunculin treatment resulted in decrease in the length of microridges (Supplementary Fig. 8b).

These data suggest that actin polymerization is required for ridge elongation at early developmental stages. In *has/apkc* mutant and MLCK-CA overexpression condition actin filaments are stable and are less dependent on actin polymerization for their maintenance. In Lgl2 overexpression condition and in 5-day-old wild-type larvae, microridges continue to depend on actin polymerization to maintain their length.

## Discussion

Whether cell polarity regulators control the formation of apical projections has remained poorly understood. Here we have used microridges in the head peridermal cells as a paradigm to show that the two key polarity regulators, aPKC and Lgl1/Lgl2 regulate formation of these actin-based apical projections.

In brush border or microvilli of the enterocytes, it has been shown that Lkb1/Par4 links cell polarity with brush border formation through a small G protein Rap2A (ref. 50). A recent report shows that knockout of *Crb3*–a vertebrate paralogue of Drosophila cell polarity gene *crumbs*–results in disorganized and shorter microvilli in mouse enterocytes^51^. Our analysis presented here show that aPKC and Lgl1/Lgl2 antagonistically control the elongation of microridges, which are laterally long actin projections. We show that functions of Lgl1/Lgl2, pNMII and the process of actin polymerization are important for building microridges in the peridermal cells. We propose that low levels of Lgl and pNMII at the apical cortex promote the fusion of short ridges to gradually build long ridges. In addition, pNMII and actin polymerization are important for ridge elongation as well as in the maintenance of longer ridges in the wild type. The function of aPKC is essential to control the levels of Lgl and pNMII at the apical cortex. In the absence of aPKC function, increased levels of pNMII and Lgl lead to actin polymerization-dependent precocious elongation of ridges. Thus, we have identified a hitherto unknown function for Lgl–a regulator of basolateral domain–at the apical cortex in zebrafish epidermal cells. We have also unravelled the functional significance of removal of Lgl from the apical domain by aPKC, a mechanism that is conserved in a variety of epithelial systems across various phyla^8^,^40^,^41^. We have been able to uncover these later and subtler functional aspects of cell polarity regulators without having profound effect on cell polarization possibly due to presence of maternal contribution of Lgl and aPKC (refs 22, 36) and functional redundancy between aPKC paralogues in zebrafish^52^.

Previous report indicates that *pen/lgl2* transcripts are contributed maternally and that zygotic expression is observed in the basal epidermis at 24 hpf (ref. 22). Our recent analysis by *in situ* hybridization shows that both *lgl1* and *lgl2* are expressed ubiquitously during epiboly stages (ReR, ID and MS, unpublished observations). Thus, Lgl1/Lgl2 might be inherited by the periderm from its precursor, the enveloping layer, and their levels are subsequently maintained by basal level expression of both the paralogues. It is intriguing that the loss-of-function of *lgl* paralogues results in such subtle phenotypes in the zebrafish epidermis at early stages. The knockdown of *lgl1*, loss-of-function of *lgl2* and the combined loss of *lgl1/lgl2* function yield quantitative reduction in the length of microridges. On the other hand, overexpression of Lgl1/Lgl2 as well as apically targeted Lgl1 results in robust increase in the ridge lengths. Thus, while Lgl function is sufficient to increase the ridge length and is necessary for their elongation to build long ridges, it might not be essential for their formation. Furthermore, though Lgl overexpression results in formation of longer ridges, it only partially recapitulates the aPKC phenotype in quantitative terms indicating that aPKC governs multiple pro-elongation factors during ridge formation. It appears that the function of ridges are of vital importance and therefore their formation is redundantly regulated by other polarity components besides Lgl. Such redundant mechanisms have been reported for maintenance of cell polarity in Drosophila epithelial tissues^53^,^54^,^55^,^56^. Thus, further investigations are required to identify additional components or polarity regulator/s that act in conjunction with the two Lgls in formation of apical microridges.

Recently, zebrafish peridermal microridges have been characterized with respect to their composition and dynamics. It has been shown that actin regulators cortactin and VASP localize to the ridges. Besides, actin polymerization and activities of Arp2/3 and PI3K signalling have been shown to be important for the maintenance of microridge length^57^. Similar to this earlier report^57^, our analysis suggests that actin polymerization is essential for the formation of actin microridges. However, in contrast to their finding^57^, we have consistently found that Blebbistatin treatment results in shortening of the ridges suggesting that pNMII has an essential role in microridge formation. Interestingly, in contrast to wild type, once formed, the longer ridges in *has/apkc* mutants become relatively stable and hence do not rely on actin polymerization for their maintenance. However, such stable structures might be detrimental for a growing epithelial system like epidermis, which would require plasticity. Hence a slower mechanism of building ridges by controlling levels of active form of myosinII and maintaining persistently low levels of Lgl must have evolved to form plastic microridges.

Microridges are present on several squamous epithelial cells. However, the regulation of their formation has remained unclear due to lack of a suitable model. This study establishes the possibility of using zebrafish epidermis as a model to unravel the molecular mechanisms involved in the formation of ridges. It is intriguing to see large variations in the patterns as well as lengths of the microridges in head peridermal cells within an embryo as well as across various embryos from the same parents or different parents while the embryos develop. These variations do not easily reveal the changes in lengths by qualitative assessment. Even after quantification, the variations and non-normal distribution of the data demand robust analysis by non-parametric statistical tests to assess the effect. Here we have employed methods consisting of (a) a new data visualization method using bean plots, (b) frequency distribution of ridge lengths in various bins, (c) analysis of means and variances for individual cells and analysis of their distribution, (d) statistical analysis using non-parametric tests such as Kruskal–Wallis test and Dunn's pairwise comparison. This methodology has allowed reliable detection of subtle differences amongst the genotypes.

To summarize, we have identified a novel regulation of microridge formation by antagonistic interactions between aPKC and Lgl. Importantly, we have identified the significance of Lgl removal from the apical domain by aPKC in vertebrate epithelial cells. Our analyses presented here suggest that formation of apical projections is under the control of mechanisms involved in establishment and maintenance of epithelial cell polarity.

## Methods

### Ethics statement

For zebrafish maintenance and experimentation, guidelines recommended by the Committee for the Purpose of Control and Supervision of Experiments on Animals (CPCSEA), Govt. of India, were followed.

### Fish strains

For experiments in wild-type embryos, the Tübingen (Tü) strain was used. Alleles *has^m567^* and *pen^t06^* were used for the mutant studies presented here^22^,^36^. Tg(cldnB:lynEGFP) line was used to visualize plasma membrane wherever necessary^37^.

### Whole-mount immunostaining

Whole-mount immunostainings in zebrafish embryos were performed as before^23^. For most of the stainings, embryos were fixed overnight in 4% PFA followed by permeabilization in methanol at −20 °C for at least overnight. For pNMII, and GFP staining, embryos were processed for staining immediately after overnight fixation in PFA. For Lgl stainings done along with phalloidin, only PFA fixation was used. For co-staining of Lgl and other markers, the overnight PFA fixed samples were post-fixed in methanol. Though there are differences in Lgl staining intensities at the apical domain in the absence and presence of methanol post-fixation, we consistently observed increased apical Lgl in *has/apkc* mutant and *apkc* morphants as compared with siblings. Notably, in *has/apkc* mutant periderm apical Lgl staining is enhanced but the siblings show near absence of the apical Lgl staining after methanol post-fixation. For staining using anti-ZO-1 antibody (1:100; ZYMED Labs, Invitrogen; 61–7,300), embryos were fixed in Dent's fixative (80:20 methanol: DMSO) overnight at −20 °C. Fixed or post-fixed embryos were washed 5 times with PBT (PBS+0.8% Triton-X 100) and incubated in 10% normal goat serum (NGS) in PBT for 3 h at room temperature. The embryos were incubated overnight with primary antibodies, diluted appropriately in 1%NGS in PBT, at 4 °C. Antibodies and their dilutions: anti-Lgl2 (1:400)^23^, monoclonal anti-E-cadherin (1:100; BD Transduction Labs; 610,182), anti-GFP (1:200; Torrey Pines Biolabs; TP401), monoclonal anti-GFP (1:40; Genei, Bangalore), anti-aPKC (1:750; Santa Cruz Biotechnology; sc-216), anti-pMyosinII (1:25; Cell Signaling Technologies; 3,671; 3675), and anti-Ezrin (1:250; Santa Cruz Biotechnology; sc-32759). Embryos were then washed five times in PBT and incubated in secondary antibodies, appropriately diluted in 1%NGS in PBT, for 4 h at room temperature. Secondary antibodies conjugated with Alexa 488 (1:250; Molecular Probes; A-11034, A-11029), Alexa 546 (1:250; Molecular probes; A-10036), Alexa 568 (1:250; Molecular probes; A11011) or Alexa 647 (1:250; Molecular Probes; A-21236, A-21245), Cy3 (Jackson Immunoresearch Labs; 115-165-144, 115-165-146) or Cy5 (1:750) (Jackson Immunoresearch Labs; 115-175-144, 115-175-146) were used. Subsequently, embryos were washed 5 times in PBT, post-fixed in 4% PFA and upgraded in glycerol for imaging using confocal microscopy. All samples required to be stained using either Alexa 488 (1:40; Molecular Probes; A12379), Rhodamine (1:40; Molecular Probes; R415) or Alexa 647 conjugated phalloidin (1:40; Molecular Probes; A22287) were fixed overnight in 4% PFA in PBS at 4 °C.

### Drug treatment

Blebbistatin (B0560, Sigma) and Latrunculin A (L5163, Sigma) were dissolved in DMSO at a stock concentration of 100 mM and 1 mM, respectively. Working dilutions of Blebbistatin and Latrunculin A, at final concentrations of 10 and 2 μM, respectively, were prepared in E3 buffer containing 1% DMSO. Embryos were treated with Blebbistatin for 1.5 h and with Latrunculin A for 30 min at 29 °C, while control embryos were incubated in 1% DMSO for the same time periods. Embryos were fixed at the appropriate stages and processed further for immunostainings.

### Morpholino, mRNA and plasmid injections

Antisense morpholino oligonucleotides (Gene Tools, LLC), were microinjected in the 1–2 cell stage embryos at the following concentrations: *lgl1*- MO*^lgl1-atg^* 5′-CCGTCTGAACCTAAACTTCATCATC-3′ (250 μM) (refs 42, 43), *lgl1*-MO*^lgl1-utr^* 5′-TGAAGCCGAATCAGAGGTAAATCAC-3′ (300 μM) (refs 42, 43), *aPKCλ*-MO aPKCλ-atg 5′-TGTCCCGCAGCGTGGGCATTATGGA-3′ (100 μM) (ref. 36). Standard control morpholino 5′-CCTCTTACCTCAGTTACAATTTATA-3′ or 5 base mismatch control morpholino 5′-CCCTCTCAACGTAAAGTTCATGATC-3′ (Gene Tools, LLC) was also used as a control for *lgl1* morpholinos. MLCK-CA construct^58^ was modified to remove (*nos*1–3′UTR) and capped mRNA was transcribed using the T7 mMessage mMachine kit (Ambion; AM1344). The mRNA was injected at a concentration of 300ng μl^−1^ in the 1–2 cell stage embryos. Xenopus GFP-Lgl2 construct^9^, pCMV LifeAct-TagRFP plasmid (ibidi; 60,102), mouse GFP-Lgl1 (ref. 33) were injected at a concentration of 100ng μl^−1^. Zebrafish Ezrin cDNA (BC139506.1) was cloned into the pCRII-TOPO vector (Invitrogen; K460001) and subcloned into the eGFP-Lgl1 vector using EcoRI (NEB; R0101S, R3101) restriction sites to make the eGFP-Ezrin-Lgl1 fusion construct. The control eGFP-Ezrin construct was made by restriction digestion using BamHI (NEB; R0136S) to release mLgl1 from the eGFP-Ezrin-Lgl1 fusion construct. The CMV-GFP-NMHC II-A plasmid used to overexpress the heavy chain of non-muscle myosin-IIA was a gift from Robert Adelstein (Addgene plasmid #11347) (ref. 59), whereas the Myl12.1-eGFP construct^60^ was a gift from C. P. Heisenberg. All constructs were injected at a concentration of 100 ng μl^−1^.

### Microscopy and image analysis

Confocal imaging was done in the head (dorsal) epidermis on the Zeiss LSM 510 Meta confocal microscope having an EC Plan-Neofluar × 40/1.30 oil-immersion objective lens or on Zeiss LSM 710 having Plan-Apochromat × 40/1.3 oil-immersion objective with an optical 2 × zoom. Image size was 1,024 × 1,024 with an averaging of 4. The pinhole values were kept as 1 Airy Unit. For time-lapse live imaging, embryos injected with pCMVLifeAct-TagRFP plasmid were dechorionated and screened for epidermal clones. Post screening, embryos were anaesthetized with 0.04% MESAB (Sigma; A5040) and mounted in 0.2% low melting agarose in plastic dishes with cover slip bottoms. Imaging was done on Zeiss LSM 5 Exciter equipped with temperature-controlled chamber using Plan-Apochromat × 63/1.40 oil objective lens with an optical × 1.5 zoom. Image size was 512 × 512 with an averaging of 4. The pinhole value for 543 laser line was kept at 106 μm and confocal stacks were captured at a slice interval of 0.411 μm. The capture rate was one frame per 12 min and played at a rate of 6 frames per second for Supplementary Videos 1, 2, 3, 4, 5, 6, 7, 8, 13, 14, 15, 16. Zeiss LSM 710 was used for capturing movies with higher temporal resolution using acquisition speed of two frames per minute and played at a rate of 6 frames per second for Supplementary Videos 9, 10, 11, 12. The Image5D plug-in from ImageJ was used for image processing and analysis of live movies.

Imaging conditions were kept uniform across most of the experiments. Occasionally, PMT gains were slightly re-calibrated to uniformly suit imaging of all the genotypes in a given set. This helps avoid saturation of a stronger signal in a particular genotype and at the same time allows detection of a weaker signal in some other genotype. For colocalization measurements involving marker pairs aPKC:F-actin, Lgl:F-actin, pNMII:F-actin and pNMII:Lgl, imaging conditions were kept same across all the genetic conditions.

To improve visualization in figure panels, some of the images were digitally processed using Adobe Photoshop CS6. However, all the quantifications have been done on raw unprocessed images.

### Estimation of microridge perimeter and surface area quantification

Embryos stained with Rhodamine phalloidin were used for quantification using ImageJ. To calculate microridge perimeter and number, the cell outline was traced in the confocal apical slice displaying microridges. Images were smoothened and thresholded such that the outlines of the ridges were clearly defined. The Analyze Particle command was used to detect the number and perimeter of the edges of microridges. Images were thresholded for a second time, if required, to detect the edge of microridges that could not be marked clearly in the first round. The Magic Wand tracing tool was used for manual detection and selection of microridges after second round of thresholding.

For each genetic condition, the perimeter quantification of microridges was done on 4–8 cells per embryo. Multiple embryos from various experimental sets were used to obtain the data. The details of the number of ridges, cells, embryos and experimental sets are provided as an excel sheet (Supplementary Data 1).

To estimate the surface area of the plasma membrane, *has/apkc* mutants in the background of Tg(cldnB:lynEGFP) line were stained with anti-GFP antibody. As published earlier^61^, to calculate the surface area, cell outlines were marked in each confocal section using the Measure Stack plug-in of ImageJ. Basolateral surface area was estimated by multiplying the perimeter of each slice by the slice thickness and adding it to the area of the first and the last slice. Area occupied by microridges in membrane folds was quantified by first obtaining the perimeter of microridges as mentioned earlier. This perimeter multiplied by the section thickness and summing this for all the confocal slices encompassing the ridges yields the membrane area for the ridges. The total surface area was estimated by adding up the basolateral surface area and the area occupied by microridges.

For surface area quantification, five cells from each embryo and a total of six embryos (*N*=6; *n*=30) were quantified for each genetic condition.

### Colocalization measurements

Localization of proteins to microridges was detected and quantified using the JACoP plugin in ImageJ. Boundaries of individual cells were marked at the apical slice displaying microridges. Five cells from each embryo and a total of eight embryos from two different experiments were used for analysis (*N*=8, *n*=40). Images were thresholded manually to mark microridges and the Pearson correlation coefficient and Manders' overlap coefficient were calculated. Manders' coefficient M1 indicates fraction of A overlapping B, while M2 indicates fraction of B overlapping A.

### Statistical analysis

Bean plots were used to visualize distribution of microridge perimeters across different genotypes. Microridge perimeter data for each genotype was collated and Bean plots along with medians were plotted in R statistical software. In addition, mean ridge length and variance was estimated for individual cells from every genotype/treatment. The distribution of the means and variances for cells from each genotype were represented using Box-whisker plots. The data analysis was done using Kruskal–Wallis one-way analysis of variance on ranks followed by pairwise multiple comparison procedures using Dunn's Method. The statistical significance of pairwise comparisons is denoted using alphabet(s) associated with each distribution in the graphs. The two distributions are not significantly different at *P*<0.05 when they are denoted by the same alphabet or when they share one alphabet in common when denoted by multiple alphabets. Extending further, the distributions are significantly different when they are not denoted by the same alphabet or do not share an alphabet in common when denoted by multiple alphabets. For example, in Fig. 3b distributions denoted by ‘a' and ‘a,e' are not statistically different when compared with each other. However, in pairwise comparisons, both these distributions are significantly different than the distributions denoted by ‘b' ‘c,d' and ‘d'. Further, though distribution denoted by ‘a' is different than that denoted by ‘c,e', the distribution ‘a,e' is not different than the one denoted by ‘c,e'. While in most of the cases the difference was considered significant at *P*<0.05, in two comparisons *P*≤0.061 was considered significant and is mentioned as such in the figure legends. SigmaPlot 13.0 was used for statistical analysis using one-way analysis of variance and for plotting box plots.

Binning of microridges into small (0–5 μm), intermediate (5–20 μm), long (20–100 μm) and very long (>100 μm) categories was done using the frequency function in EXCEL. Microridge frequencies of a given category were expressed as a percentage of the total number of microridges in a cell. The data were plotted against developmental time or genotypes using Microsoft EXCEL. Histograms for comparison of membrane surface area and box and whisker graphs for colocalization coefficients were plotted using Microsoft Excel. Two-tailed Student's *t*-tests for pairwise comparison of means using Microsoft EXCEL was conducted. A significance level of 0.05 was used as cutoff.

### Data availability

The authors declare that the data supporting the findings of this study are available within the article and its Supplementary Information files.

## Additional information

**How to cite this article:** Raman, R. *et al*. aPKC regulates apical localization of Lgl to restrict elongation of microridges in developing zebrafish epidermis. *Nat. Commun.* 7:11643 doi: 10.1038/ncomms11643. (2016).

## Supplementary Material

Supplementary Information Supplementary Figures 1-9, Supplementary Note 1 and Supplementary References

Supplementary Data 1 This Excel file contains the details about number of embryos, number of cells, number of ridges, etc analysed for each experiments that has been reported in the paper.

Supplementary Movie 1 Time-lapse movie of microridges during 19-22hpf in wild type peridermal cells expressing LifeActRFP. Acquisition rate = 1 frame/12mins; played at 6 frames/second.

Supplementary Movie 2 Time-lapse movie of microridges from the marked area of 100X75 pixels in Supplementary Video 1 and zoomed 3.2 times.

Supplementary Movie 3 Video of microridges in wild type peridermal cells from 27-30hpf. Acquisition rate=1 frame/12mins; played at 6 frames/second.

Supplementary Movie 4 Time-lapse video of microridges taken from the marked area of 100X75 pixels from Supplementary Video 3 and zoomed 3.2 times.

Supplementary Movie 5 Time-lapse imaging of microridges in *apkc* morphant peridermal cells from 19-22hpf acquired at 1 frame/12mins and played at 6 frames/second.

Supplementary Movie 6 Time-lapse imaging of microridges from area of 100X75 pixels in Supplementary Video 5 and zoomed 3.2 times.

Supplementary Movie 7 Time-lapse analysis of microridges in *has/apkc* mutant peridermal cells from 27-30hpf acquired at 1 frame/12mins and played at 6 frames/second.

Supplementary Movie 8 Time lapse Movie of microridges in *has/apkc* mutant peridermal cells from 27-30hpf, cropped from area of 100X75 pixels from Supplementary Movie 7 and zoomed 3.2 times.

Supplementary Movie 9 Time-lapse video of wild type microridges at 21hpf acquired at 2 frames/min and played at 6 frames/second.

Supplementary Movie 10 Wild type microridges cropped from area of 100X75 pixels in Supplementary Video 9 and zoomed 3.2 times.

Supplementary Movie 11 Time-lapse analysis of *apkc* morphant microridges at 21hpf. Acquisition=2 frames/min; played at 6 frames/second. Arrows indicate the fusion events.

Supplementary Movie 12 Time-lapse video of *apkc* morphant microridges taken from area of 100X75 pixels from Supplementary Video 11 and zoomed 3.2 times. Arrows indicate the fusion events.

Supplementary Movie 13 Time-lapse video of microridges in wild type peridermal cells from 23-30hpf acquired at 1 frame/12mins and played at 6 frames/seconds.

Supplementary Movie 14 Time-lapse movie of microridges in wild type peridermal cells from 23-30hpf, cropped from area of 100X75 pixels from Supplementary Video 13 and zoomed 3.2 times.

Supplementary Movie 15 Time-lapse analysis of microridges in *lgl1* morphant peridermal cells from 23-30hpf. The movie is acquired at 1 frame/12mins and played at 6 frames/second.

Supplementary Movie 16 Time-lapse video of microridges cropped from an area of 100X75 pixels from Supplementary Video 15 and zoomed 3.2 times.
