# Supplementary material for: aPKC regulates apical localization of Lgl to restrict elongation of microridges in developing zebrafish epidermis
Source: Nat Commun. 2016 Jun 1;7:11643. doi: 10.1038/ncomms11643 (PMC4895443; doi:10.1038/ncomms11643)
Supplement: Supplementary Information — Supplementary Figures 1-9, Supplementary Note 1 and Supplementary References [file ncomms11643-s1.pdf]

Supplementary Figures

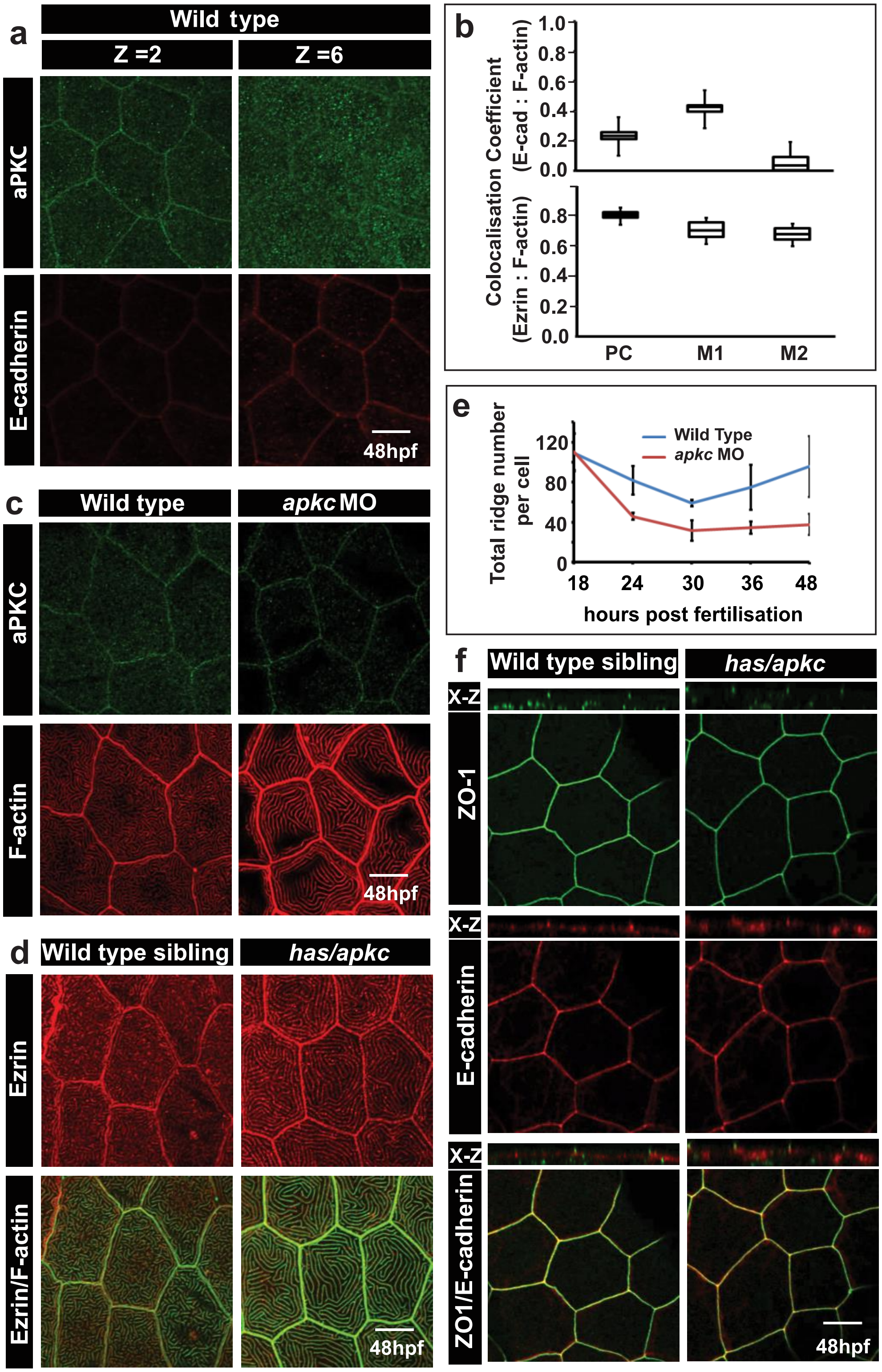

Supplementary Figure 1. aPKC localises to the apical domain in peridermal cells but does not play a role in formation of cellular junctions.

Immunostaining using anti-aPKC and E-cadherin antibodies (a) in wild-type embryos at 48hpf. Z=2 and Z=6 indicate the 2nd (apical) and the 6th (basolateral) confocal sections, respectively,

from the apical side. Box-whisker plot for co-localisation coefficients (b; top) between E-cadherin and F-actin by Pearson's method (PC) and Manders' overlap of E-cadherin with F-actin (M1) and F-actin with E-cadherin (M2). Similar Pearson's analysis (PC) between Ezrin and F-actin (b; bottom) and Manders' overlap of Ezrin with F-actin (M1) and F-actin with Ezrin (M2) at 48hpf in wild-type embryos. Immuno-localisation using anti-aPKC antibody and phalloidin (c) in wild-type and *apkc* morphants at 48hpf. Immuno-localisation using anti-Ezrin antibody and phalloidin (d) in wild-type and *has/apkc* mutants at 48hpf. Quantification of total number of ridges (e) per peridermal cell during various developmental time points, based on phalloidin stainings performed in wild-type and *apkc* morphant embryos. Confocal images of stainings for ZO1 and E-cadherin in wild-type embryos and *has/apkc* mutants at 48hpf (f). X-Z are orthogonal sections.

Basolateral marker E-cadherin does not localise to apical microridges (b) and serves as a negative control with Pearson coefficient=0.26±0.65. The E-cadherin antibody generates a few background speckles in the apical domain. Since some of these speckles fall on F-actin, it yields high Manders overlap coefficient of M1=0.413±0.14, which is misleading. However, F-actin shows very low overlap with E-cadherin background speckles yielding a very low Manders' coefficient of M2=0.005±0.004. Ezrin serves as a positive control for correlation coefficient analysis. A major fraction of Ezrin localises with actin (b,d) giving rise to Pearson coefficient of 0.813±0.04. Similarly, Manders' coefficients of Ezrin with F-actin (M1=0.73±0.09) and F-actin with Ezrin (M2=0.68±0.08) are high, reflecting significant overlap between Ezrin and F-actin (b,d).

Scale bar = 10 µm. Abbreviations: PC- Pearson's coefficient; M1 and M2- Manders overlap coefficients.

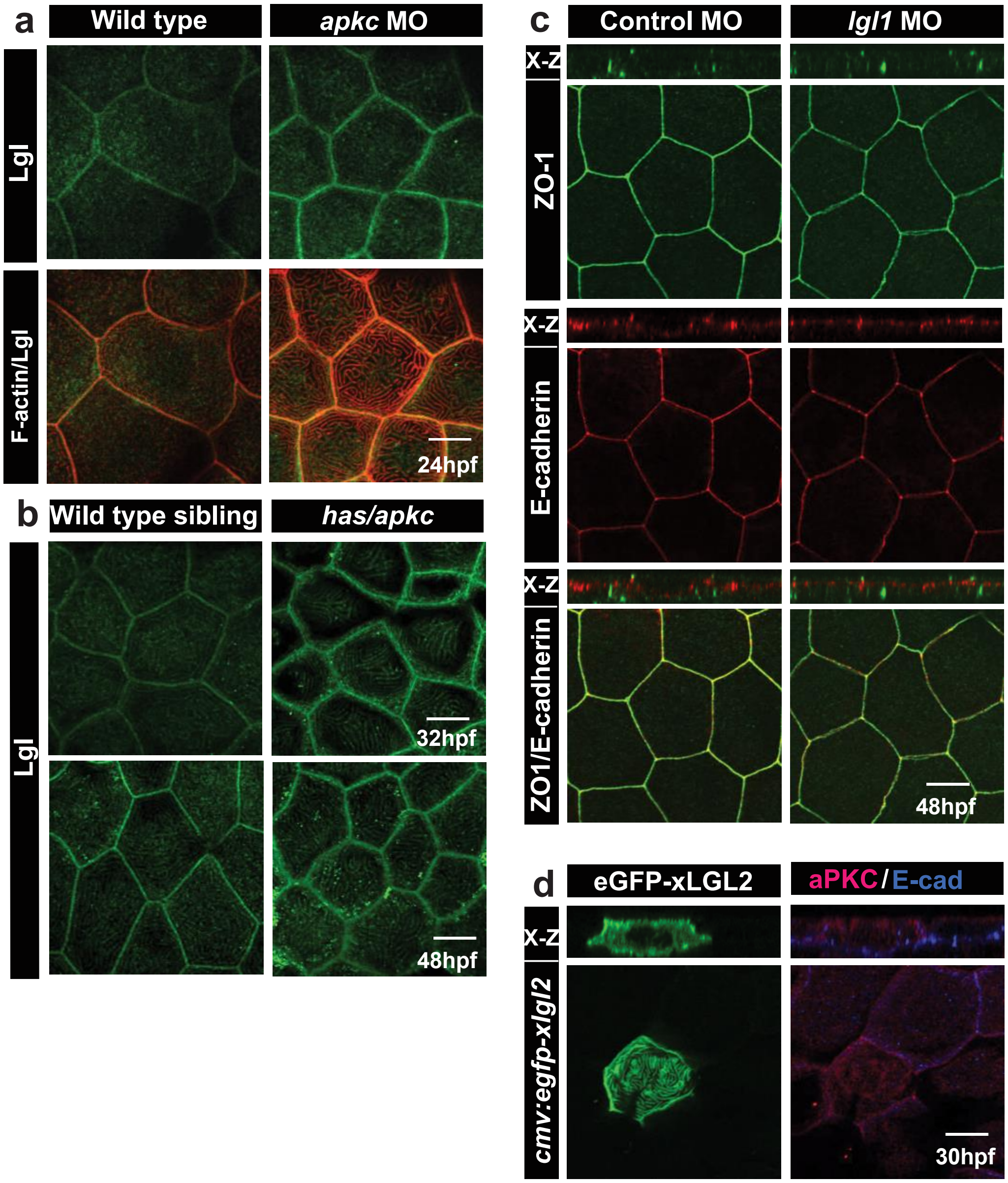

Supplementary Figure 2. Lgl co-localises with F-actin at microridges and exhibits temporal increase in its levels in aPKC deficient embryos. Lgl1 deficiency or Lgl2 overexpression does not perturb the overall polarity in the peridermal cells.

Immuno-colocalisation using anti Lgl2 antibody and phalloidin (a) in wild-type and aPKC deficient embryos at 24hpf. Lgl2 staining in wild-type sibling and in *has/apkc* mutants at 32 and 48hpf (b). Confocal images along with orthogonal (X-Z) sections of ZO1 and E-cadherin stainings in wild-type embryos and *lgl1* morphants at 48hpf (c). Immuno-localisation using anti aPKC and anti E-cadherin antibodies in clones expressing *cmv:egfp-xlgl2* construct (d) at 30hpf.

Scale bar = 10µm.

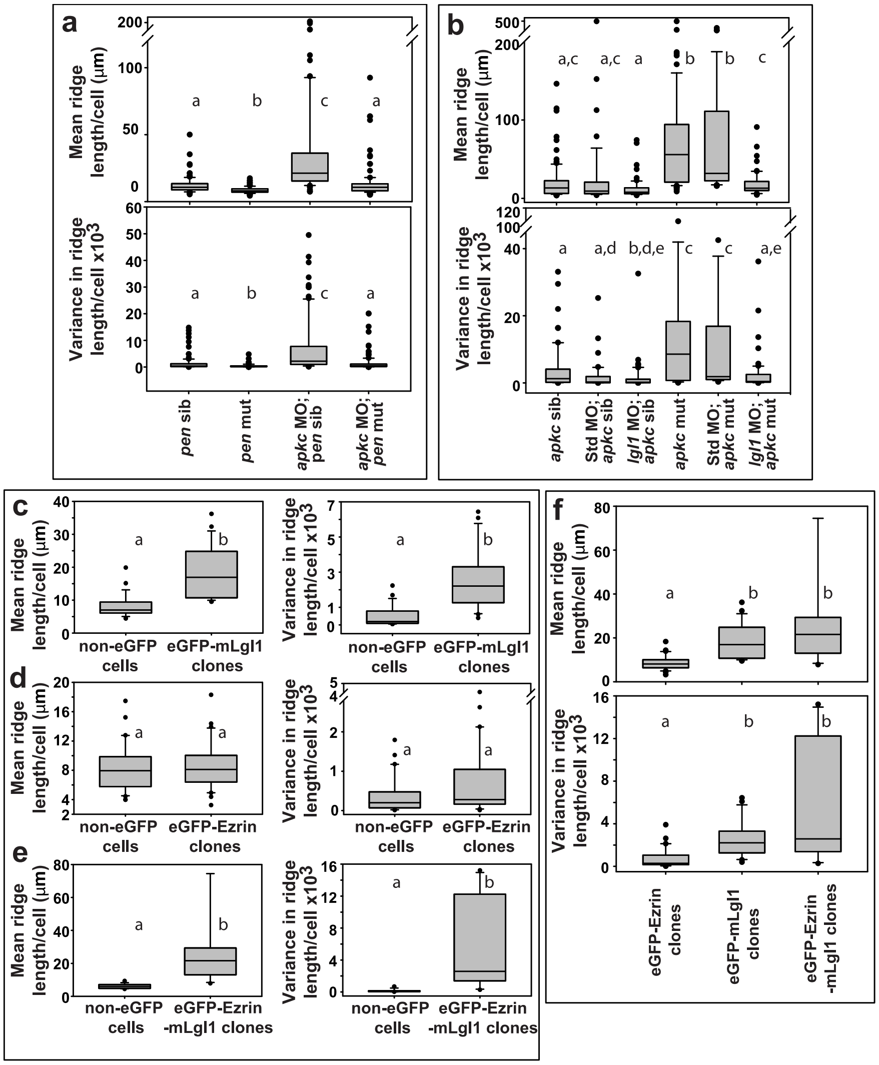

Supplementary Figure 3. Estimation of means and variances of microridge length per cell indicates that aPKC controls Lgl levels at the apical domain to restrict the microridge length.

Estimation of mean and variance in ridge length for individual cells and comparing the distributions of means and variances in *pen/lgl2* sibling, *pen/lgl2* mutant, *apkc* MO embryos and *apkc* MO;*pen/lgl2* embryos (a) and in *apkc* sibling, std control MO;*apkc* sibling, *lgl1MO*;*apkc* sibling*, has/apkc* mutant, std control MO;*has/apkc* mutant and *lgl1 MO*;*has/apkc* mutant embryos (b) using box-whisker plots. Similar analysis for clones expressing eGFP-mLgl1, eGFP-Ezrin and eGFP-Ezrin-mLgl1 and their corresponding non-GFP controls (c-e), using box-whisker plots. In (f) comparison between means and variances for eGFP-Ezrin, eGFP-mLgl1 and eGFP-Ezrin-mLgl1clones is shown.

The distributions sharing the same alphabet do not differ significantly (Dunn's multiple comparisons test, p-value < 0.05). The two distributions in (b; top), *lgl1*MO;*apkc* sib and *lgl1* MO; *apkc* mut are significantly different at p=0.057.

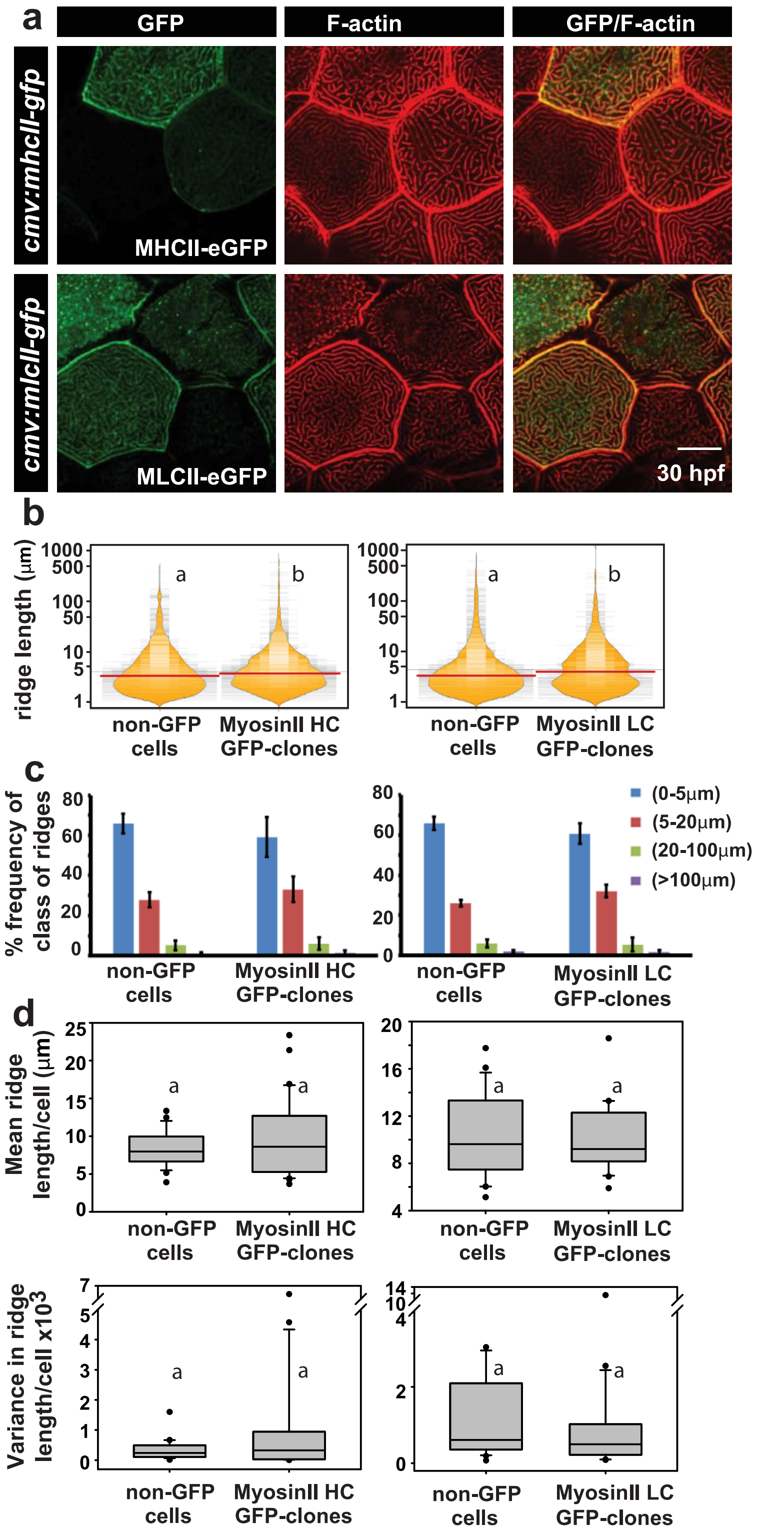

Supplementary Figure 4. Over-expression of heavy and light chains of MyosinII does not cause significant changes in ridge lengths.

Confocal images of wild-type embryos injected with *cmv:mhcII-gfp* or *cmv:mlcII-gfp* and stained for GFP and F-actin at 30 hpf (a). Visualisation of the distribution of the ridge lengths and

medians of the clones over-expressing *cmv:mhcII-gfp* or *cmv:mlcII-gfp* and the neighbouring non-GFP cells using bean plots (b). The percentage frequency distribution of ridges in short (0-5 µm), intermediate (5-20 µm), long (20-100 µm) and very long (>100 µm) categories (c), as well as estimation of mean and variance in ridge length for individual cells and comparison of their distributions (d) using box-whisker plots. Quantifications in (b), (c) and (d) are based on embryos injected with *cmv:mhcII-gfp* or *cmv:mlcII-gfp* and stained for GFP and F-actin at 30 hpf.

Distributions represented with different alphabets show significant difference in median values at $p<0.05$ by pairwise multiple comparison procedures using Dunn's Method. Error bars in (c) are for the standard deviation. Scale bar =10 µm. Abbreviations: MHCII: MyosinII heavy chain; MLCII: MyosinII Light chain.

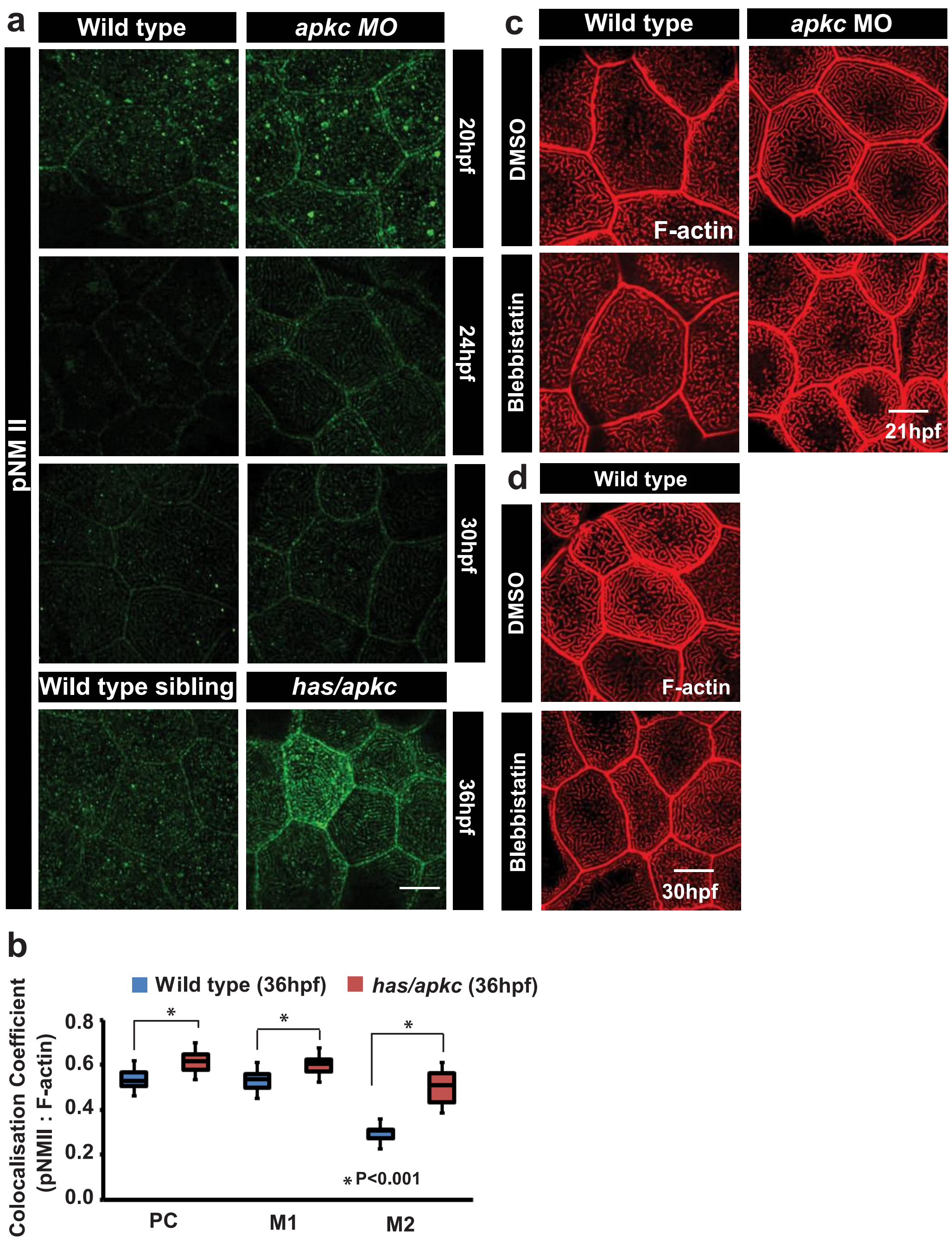

Supplementary Figure 5. MyosinII activity (pNMII levels) is higher in aPKC deficient embryos and is required for elongation of ridges in wild-type as well as in *apkc* morphants.

pNMII staining (a) in wild-type and *apkc* morphant embryos at 20, 24 and 30hpf and in *has/apkc* mutants at 36hpf. Box-whisker plot for colocalisation coefficients (b) of F-actin and pNMII by Pearson's methods (PC) and Manders' overlap coefficient of pNMII with F-actin (M1) and F-actin with pNMII (M2) at 36 hpf in wild-type and *has/apkc* mutant embryos. Wild-type and *apkc* morphants (*apkc* MO) treated with 10µM Blebbistatin for 1.5h, fixed at 21hpf (c) and at 30hpf (d) followed by phalloidin staining to visualise F-actin.

Probability values “P” are for the student’s t–test. Asterisk in (b) indicate significant difference at $p<0.001$. Scale bars correspond to 10 µm. Abbreviation: pNMII- phospho Non muscle-MyosinII.

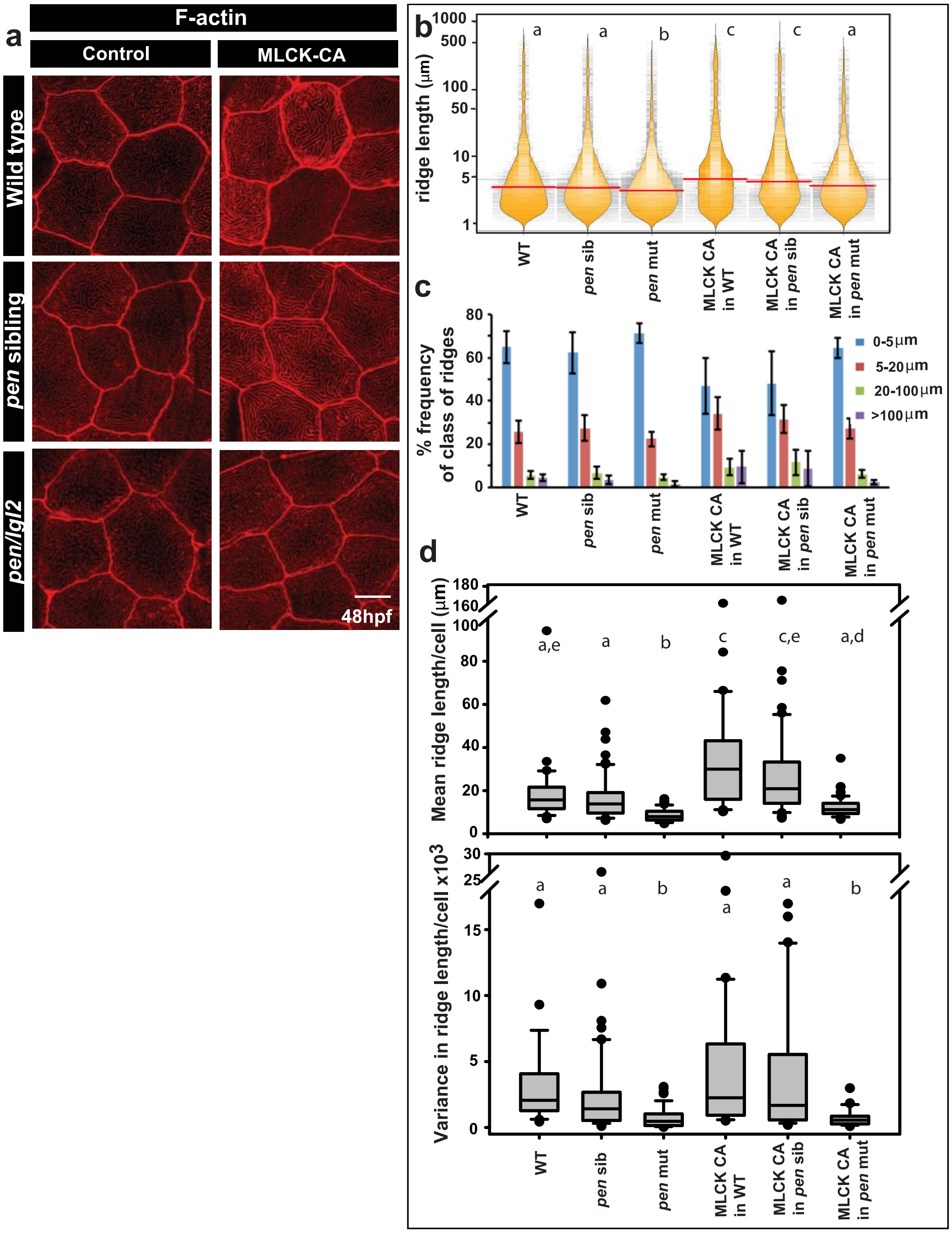

Supplementary Figure 6. Over-expression of MLCK-CA does not give rise to longer ridges in *pen/lgl2* mutants.

Confocal microscopy analysis of the peridermal cells in given genotypes in either uninjected (control) or MLCK-CA injected embryos stained with phalloidin (a). Graphical representation of the distribution of ridge lengths and corresponding medians in various genotypes using bean plot (b). A bar graph showing percentage frequency distribution of ridges in short (0-5 µm), intermediate (5-20 µm), long (20-100 µm) and very long (>100 µm) categories (c). Representation of cell wise means and variances of ridge lengths in various genotypes using box-whisker plot (d).

In (b) and (d) the distributions sharing the same alphabet do not differ significantly (Dunn's multiple comparisons test, p-value < 0.05). Error bars in (c) are for the standard deviation. Scale bar in (a) is equivalent to 10 µm.

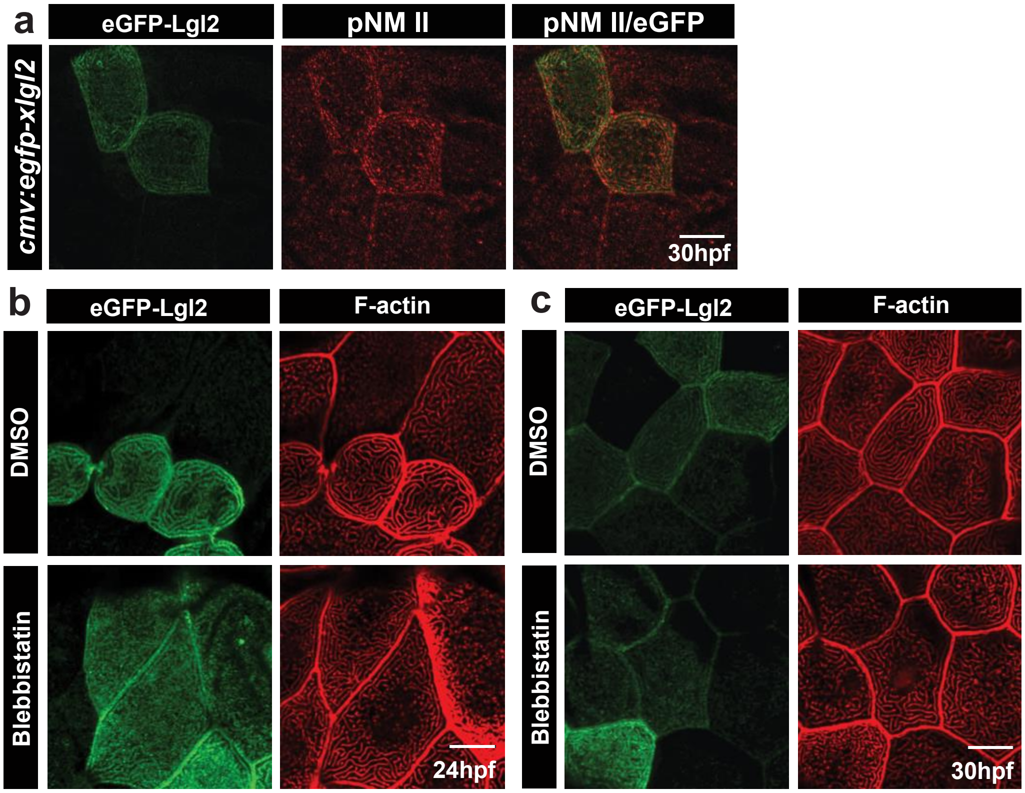

Supplementary Figure 7. Lgl over-expression results in increased pNMII levels; MyosinII activity is essential for the manifestation of long ridge phenotype in cells over-expressing Lgl.

Confocal images of wild-type embryos injected with *cmv:egfp-xlgl2* and stained for GFP and pNMII at 30 hpf (a). eGFP-xLgl2 clones (b,c) treated with 10μM Blebbistatin for 1.5h, fixed at (b) 24hpf and (c) 30hpf and stained using phalloidin to visualise F-actin.

Scale bar corresponds to 10 μm.

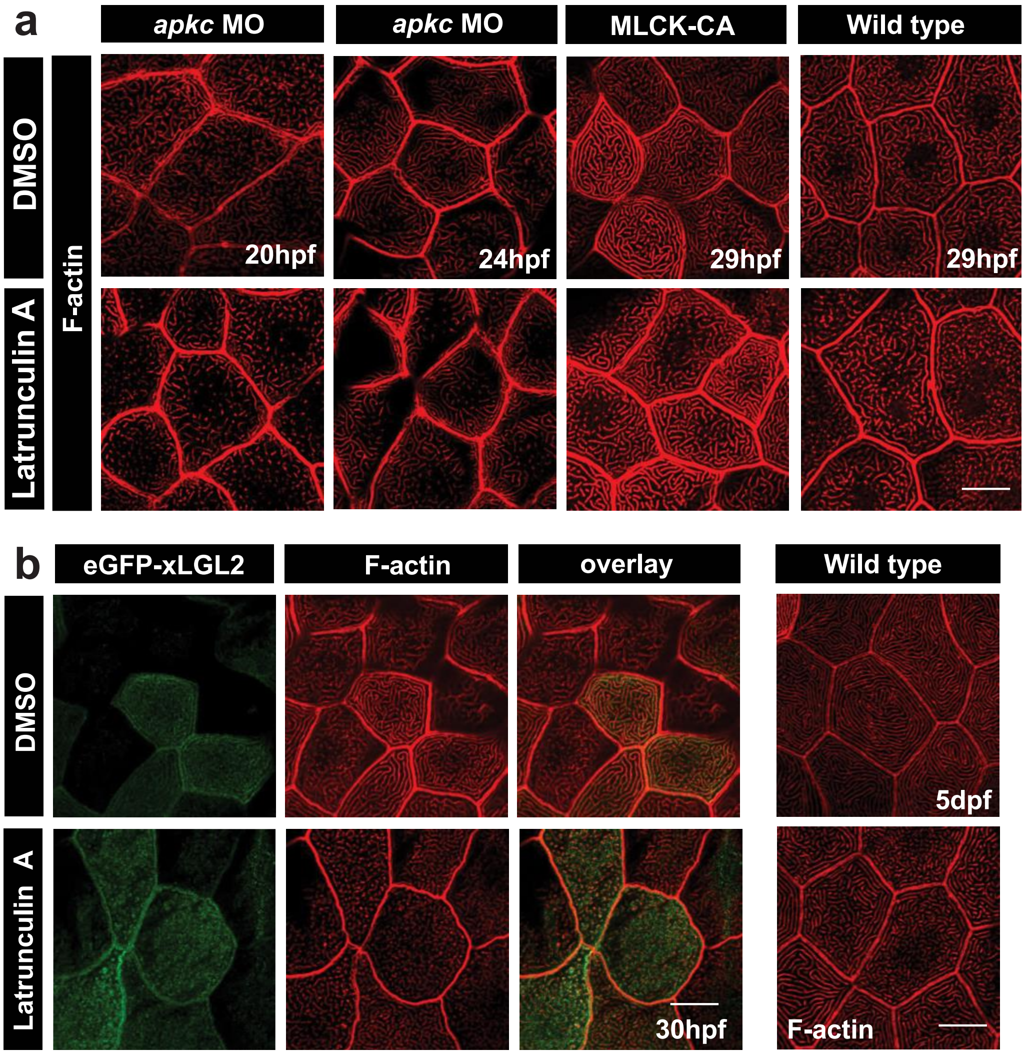

Supplementary Figure 8. F-actin polymerisation is required during ridge elongation phases in various genetic backgrounds.

Peridermal cells in *apkc* morphant at 20 and 24hpf; MLCK-CA injected and wild-type embryos at 29hpf (a), eGFP-xLgl2 clones at 30hpf and wild-type larvae at 5dpf (b), treated with 2µM Latrunculin A for 30 minutes, fixed and stained using phalloidin to visualise F- actin.

Scale bar corresponds to 10 µm.

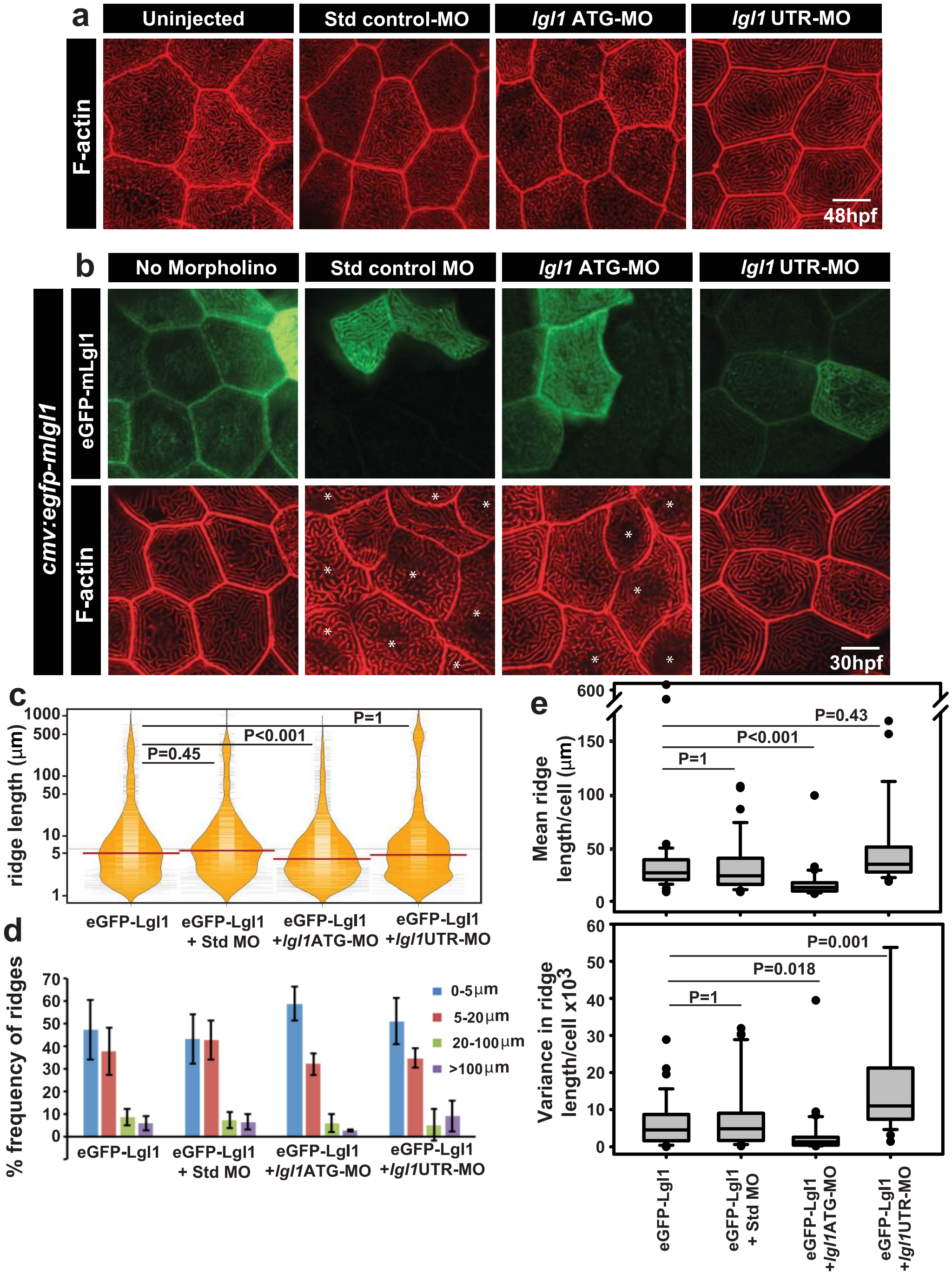

Supplementary Figure 9. Assessment of the specificity of *lgl1* morpholinos used in this study.

Confocal images of microridge F-actin stained by phalloidin in un-injected embryos and embryos injected with standard control morpholino (Std control MO), *lgl1*ATG morpholino (*lgl1*ATG-MO) and *lgl1*UTR morpholino (*lgl1*UTR-MO) at 48hpf (a).

Analysis of ridge lengths by F-actin staining in peridermal clones expressing eGFP-mLgl1 in embryos co-injected with the mentioned morpholino (or no morpholino) and a vector driving

expression of *egfp-mlgl1* under CMV promoter (b). Quantification of ridge lengths in the clones and visualisation of the length distributions and the respective medians in various genetic conditions by bean plot (c). The percentage frequency distribution of ridges in short (0-5 µm), intermediate (5-20 µm), long (20-100 µm) and very long (>100 µm) categories for the given genetic conditions (d). Estimation of cell wise means and variances of ridge lengths for each genetic condition and their representation by box-whisker plot (e).

In (c) and (e), the horizontal black lines and associated probability values (P) represent the comparison by Dunn's multiple comparisons. $P<0.05$ is considered statistically significant. In (d) Standard deviation is shown by error bars. Scale bars in (a,b) are equivalent to 10µm. The asterisks in (b) depict the cells that do not express eGFP-mLgl1. Abbreviation: Std: standard; MO: morphant/morpholino, mLgl1: mouse Lgl1

Supplementary Note 1

We tried previously published two morpholinos for knocking down *lgl1*[42,43]. One of the morpholinos is designed against the translational start site (*lgl1*ATG morpholino or *lgl1*-MO$^{lgl1\text{-}atg}$) and the second morpholino targets the 5'UTR site (*lgl1*UTR morpholino or *lgl1*-MO$^{lgl1\text{-}utr}$)[1,2]. Both the morpholinos recapitulated the previously published morphological phenotype characterised by reduced brain and mild pericardial edema[1,2]. At the cellular level, the ATG morpholino showed a shorter microridge phenotype in the periderm whereas the UTR morpholino showed longer ridges, which were frequently interconnected (Supplementary Fig.9a). Since such differences in the phenotypes could be due to tissue specific off target effects, we set out to validate the specificity of the two morpholinos. We combined the clonal over-expression of eGFP tagged mLgl1 (eGFP-mLgl1) with knockdown of *lgl1* using the two morpholinos in question. We reasoned that Lgl1 over-expression phenotype characterized by long ridges at 30hpf (main text, Figure 6a) should revert back towards comparatively shorter ridges upon reduction of Lgl1 levels by morpholino knockdown. Confocal microscopy combined with ridge length quantification in the clones and four ways of data analyses (see the main text for details) revealed that the clones over-expressing eGFP-mLgl1 showed longer ridges. Co-injection of standard control morpholino as well as UTR morpholino did not rescue the long ridge phenotype observed upon Lgl1 overexpression. The ATG morpholino rescued the Lgl1 over-expression phenotype resulting in significantly shorter ridge length as revealed by the significant shift of the median towards a shorter ridge length, increase in the frequency of short ridges and decrease in the mean ridge-length per cell as well as decrease in the variance in ridge lengths per cell (Supplementary Fig.9b-e). We concluded that the ridge phenotype of ATG morpholino is specific and used it for the analyses.

Supplementary References

1. Clark, B.S. *et al.* Loss of Llgl1 in retinal neuroepithelia reveals links between apical domain size, Notch activity and neurogenesis. *Development (Cambridge, England)* **139**, 1599-1610 (2012).
2. Hava, D. *et al.* Apical membrane maturation and cellular rosette formation during morphogenesis of the zebrafish lateral line. *Journal of cell science* **122**, 687-695 (2009).
